# Luman contributes to brefeldin A-induced prion protein gene expression by interacting with the ERSE26 element

**DOI:** 10.1038/srep42285

**Published:** 2017-02-13

**Authors:** Marc-André Déry, Andréa C. LeBlanc

**Affiliations:** 1 Lady Davis Institute for Medical Research, Sir Mortimer B. Davis Jewish General Hospital, Montreal, Quebec, Canada; 2 Department of Neurology and Neurosurgery, McGill University, Montreal, Quebec, Canada

## Abstract

The cellular prion protein (PrP) is essential for transmissible prion diseases, but its exact physiological function remains unclear. Better understanding the regulation of the human prion protein gene (*PRNP*) expression can provide insight into this elusive function. Spliced XBP1 (sXBP1) was recently shown to mediate endoplasmic reticulum (ER) stress-induced *PRNP* expression. In this manuscript, we identify Luman, a ubiquitous, non-canonical unfolded protein response (UPR), as a novel regulator of ER stress-induced *PRNP* expression. Luman activity was transcriptionally and proteolytically activated by the ER stressing drug brefeldin A (BFA) in human neurons, astrocytes, and breast cancer MCF-7 cells. Over-expression of active cleaved Luman (ΔLuman) increased PrP levels, while siRNA-mediated Luman silencing decreased BFA-induced *PRNP* expression. Site-directed mutagenesis and chromatin immunoprecipitation demonstrated that ΔLuman regulates *PRNP* expression by interacting with the ER stress response element 26 (ERSE26). Co-over-expression and siRNA-mediated silencing experiments showed that sXBP1 and ΔLuman both up-regulate ER stress-induced *PRNP* expression. Attempts to understand the function of *PRNP* up-regulation by Luman excluded a role in atorvastatin-induced neuritogenesis, ER-associated degradation, or proteasomal inhibition-induced cell death. Overall, these results refine our understanding of ER stress-induced *PRNP* expression and function.

Cellular prion protein (PrP) plays a fundamental role in the development of prion diseases. PrP is necessary for prion infection and its levels influence the progression of prion disease^1^,^2^,^3^. In non-infectious conditions, PrP has beneficial effects. PrP is involved in synaptic transmission^4^, cell signaling^5^, cell adhesion^6^, white matter maintenance^7^, hematopoietic differentiation^8^, and protection against oxidative stress^9^, endoplasmic reticulum (ER) stress^10^, and Bax-mediated cell death^11^,^12^,^13^,^14^,^15^. Furthermore, PrP has been closely linked to cancer resistance, tumorigenesis, and proliferation (reviewed in ref. 16). Despite these important roles of PrP in maintaining tissue homeostasis, the underlying molecular mechanisms regulating prion protein gene (*PRNP*) expression are not well defined. A better understanding of the regulation of *PRNP* expression will help clarify the physiological purpose of PrP, and is necessary to harness the roles of PrP in disease and tissue homeostasis.

The human *PRNP* is composed of a large intron flanked by two exons^17^. The *PRNP* promoter region is devoid of a TATA box, but contains a CpG island characteristic to housekeeping genes. Consistent with this feature, the *PRNP* is broadly expressed in the human body^18^. The expression of the *PRNP* is regulated by p53^19^, oxygen levels^20^,^21^,^22^, and copper exposure^23^. In addition, nerve growth factor increases *PRNP* promoter activity and *PRNP* mRNA levels in the developing brain^24^,^25^. The *PRNP* promoter contains several elements, including the heat shock (HSE), nuclear factor IL-6 (NF-IL6), specificity protein 1 (SP1), and muscle-specific factor (MyoD) elements^26^. Recently, four functional endoplasmic reticulum stress response elements (ERSE) were identified in the *PRNP* promoter region and *PRNP* expression was shown to be up-regulated by ER stress^10^.

ER stress triggers the activation of the unfolded protein response (UPR), a signaling cascade that attenuates overall translation, up-regulates the expression of genes necessary to restore adequate protein folding, promote ER-associated degradation (ERAD) of misfolded proteins, or trigger the apoptosis of cells under unresolvable ER stress. The UPR can be activated *via* three canonical pathways: the ER transmembrane sensors protein kinase RNA-like endoplasmic reticulum kinase (PERK), inositol-requiring enzyme 1α (IRE1α), and activating transcription factor 6α (ATF6α). PERK activation leads to eIF2α phosphorylation, an event that attenuates overall translation, but promotes the translation of the activating transcription factor 4 (ATF4)^27^. Activation of IRE1α enables the splicing of X-box binding protein 1 (XBP1) mRNA, causing a frame shift necessary to the translation of the functional spliced XBP1 (sXBP1) transcription factor^28^. Lastly, ATF6α is expressed as an ER-resident transmembrane protein that, upon ER stress, progresses to the Golgi apparatus, where it undergoes a proteolytic cleavage that releases its N-terminal cytosolic region, the active cleaved ATF6α (ΔATF6α) transcription factor^29^. Of these three factors, sXBP1 and ΔATF6α, but not ATF4, are linked to *PRNP* expression during ER stress^10^. Indeed, sXBP1 and ΔATF6 over-expression increases *PRNP* promoter activity, and both factors bind the *PRNP* promoter in ER stressed cells^10^. However, the siRNA-mediated silencing of ATF6α does not influence ER stress-induced PrP levels, and XBP1 silencing attenuates, but does not abolish, ER stress-induced *PRNP* expression in MCF-7 cells^10^,^30^. This indicates that neither factor is fully sufficient for ER stress-induced *PRNP* expression, and suggests the participation of additional alternative transcriptional UPR mediators.

The OASIS family of transcription factors is emerging as a group of novel, specialized, tissue-specific UPR regulators (reviewed refs 31 and 32). The OASIS family is constituted of OASIS/CREB3L1, BBF2H7/CREB3L2, CREBH/CREB3L3, AIbZIP/CREB3L4/CREB4 and Luman/LZIP/CREB3 family members. All members share bZIP and ER transmembrane domains. However, OASIS family members are differentially expressed, activated by distinct stimuli, and bind to different response elements^31^. In addition, most OASIS family members show high tissue specificity, with the exception of Luman, which is ubiquitously transcribed^33^. Like the other OASIS family members and ATF6α, Luman is an ER localized transmembrane protein. During ER stress, Luman undergoes regulated intramembrane proteolysis^34^,^35^, a process mediated by Golgi-resident proteases that release the cytosolic N-terminal portion of the protein. Active cleaved Luman (ΔLuman) then translocates to the nucleus, where it interacts with *cis*-acting promoter elements. ΔLuman binds cAMP- response element (CRE), CCAAT/enhancer binding protein (C/EBP) element^33^, endoplasmic reticulum stress response element II (ERSEII)^36^, and unfolded protein response element (UPRE)^37^. Ultimately, Luman promotes the expression of ERAD-associated genes, such as *EDEM*^37^, *HERPUD1*^36^, *Canx* and *Ubxn4*^38^, and of cholesterol metabolism regulators *Insig1, Insig2* and *Srebp1*^38^.

The objective of this study was to investigate the contribution of the OASIS family members and, more specifically, the Luman transcription factor to the regulation of *PRNP* expression by ER stress. Luman was transcriptionally and proteolytically activated by brefeldin A (BFA) in primary human central nervous system (CNS) neurons and astrocytes, and in MCF-7 breast carcinoma cells. Over-expression of ΔLuman increased *PRNP* mRNA and promoter activity, and PrP levels, and siRNA-mediated silencing of Luman reduced BFA-induced *PRNP* expression. Mutation of the ERSE26 element attenuated ΔLuman-mediated increase in *PRNP* promoter activity, and ΔLuman binding to the *PRNP* promoter ERSE26 region was confirmed by chromatin immunoprecipitation. Functionally, we exclude a putative role of Luman-mediated *PRNP* expression in (1) ERAD of misfolded proteins, (2) protecting against proteasomal inhibition-induced apoptosis or (3) atorvastatin-induced neuritogenesis.

Collectively, these results indicate that Luman contributes to BFA-induced *PRNP* expression by interacting with the ERSE26 element.

## Results

### BFA-induced ER stress increases transcription and N-terminal cleavage of Luman

**Figure 1:**
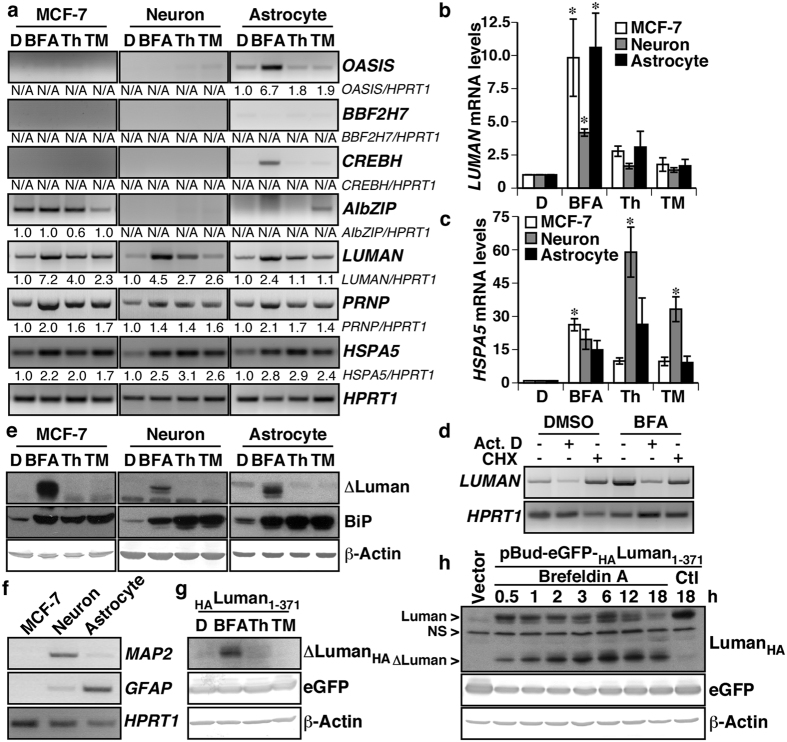
BFA-induced ER stress increases transcription and N-terminal cleavage of Luman. **(a)** Ethidium bromide stained agarose gel showing *OASIS, BBF2H7, CREBH, AIbZIP, LUMAN, PRNP, HSPA5* and *HPRT1* RT-PCR amplification products from MCF-7 cells (*n* = 4), neurons (*n* = 7) and astrocytes (*n* = 5) treated with DMSO (D, 0.1%), or 5 μg/mL of brefeldin A (BFA), thapsigargin (Th) or tunicamycin (TM) for 18 h. The fold increase relative to DMSO of band intensity normalized to *HPRT1* is indicated. (**b,c**) Relative increase in *LUMAN* (**b**) or *HSPA5* (**c**) mRNA levels assessed by qPCR 18 h after DMSO, BFA, Th or TM treatments (5 μg/mL). Data represent the mean ± SEM of three experiments, analysed using a one-way ANOVA followed by a Dunnett post-hoc test *p < 0.05 compared to DMSO. **(d)** Ethidium bromide stained agarose gel showing *LUMAN* and *HPRT1* RT-PCR amplification products from MCF-7 cells treated with DMSO or BFA (5 μg/mL) for 18 h, in the presence of 1 μg/mL actinomycin D (Act. D) or 20 μg/mL cycloheximide (CHX)). (**e**) Western blot analysis of ΔLuman, BiP, and β-Actin protein levels from MCF-7 cells or primary human neuron and astrocytes treated with 0.1% DMSO or 5 μg/mL of BFA, Th or TM for 18 h. (**f**) Ethidium bromide stained agarose gel showing *MAP2, GFAP* and *HPRT1* RT-PCR amplification products from MCF-7 cells, neurons and astrocytes^60^. (**g**) Western blot analysis of HA-tagged ΔLuman, eGFP and β-Actin protein levels from HEK293T transfected with an N-terminal HA-tagged full length Luman treated with 0.1% DMSO or 5 μg/mL of BFA, Th or TM for 18 h. (**h**) Time course assessment of Luman cleavage in HEK293T transfected with N-terminal HA-tagged full length Luman, and treated with BFA (5 μg/mL) or DMSO (Ctl, 0.1%). Vector designates HEK293T cells transfected with the pBud-eGFP vector. Full-length images of blots and gels are presented in Supplementary Information.

To identify which members of the OASIS family could contribute to ER stress-induced *PRNP* expression, *OASIS, BBF2H7, CREBH, AIbZIP*, and *LUMAN* transcript levels were assessed by RT-PCR in breast carcinoma MCF-7 cells, human primary neurons and astrocytes treated with the ER stressing drugs BFA, Th or TM (Fig. 1a). As previously observed, all three ER stressors increased *PRNP* mRNA levels. *OASIS* mRNA levels were only increased by BFA in astrocytes. Levels of *BBF2H7* and *CREBH* were undetectable, or very low, in the three cell types and seemed unaffected by ER stress, with the exception of a salient *CREBH* increase in astrocytes treated with BFA. *AIbZIP* was very weakly detected in neuronal preparations. TM increased *AIbZIP* levels in astrocytes, but reduced them in MCF-7 cells. *LUMAN* transcripts were detected in the three cell types, and BFA treatment clearly increased *LUMAN* mRNA levels in MCF-7 cells, neurons and astrocytes. However, Th and TM treatments only caused modest and inconsistent increases in *LUMAN* mRNA levels. To clarify these results, the induction of *LUMAN* mRNA by ER stress was assessed by quantitative PCR, and showed a significant *LUMAN* mRNA increase in MCF-7, neuronal and astrocytic cultures treated with BFA, but not with Th and TM (Fig. 1b). Amplification of BiP mRNA (*HSPA5*) by RT-PCR (Fig. 1a) and quantitative PCR (Fig. 1c) controlled for induction of ER stress by BFA, Th and TM treatments, and the housekeeping gene *HPRT1* was used as a control for overall mRNA levels. To confirm that the induction of Luman mRNA levels by BFA was due to an increase in *LUMAN* transcription, MCF-7 cells were treated with BFA in the presence of the transcription inhibitor actinomycin D or of the translation inhibitor cycloheximide. BFA treatment increased *LUMAN* mRNA levels, and actinomycin D co-treatment attenuated *LUMAN* mRNA levels of BFA- and DMSO-treated cells (Fig. 1d). Cycloheximide treatment did not significantly influence BFA-induced *LUMAN* mRNA, but caused a small *LUMAN* mRNA increase in the DMSO control condition, as observed previously for other genes^39^. The housekeeping gene *HPRT1* controlled for overall mRNA levels.

Contrary to human primary neurons and astrocytes, MCF-7 cells are readily available, transfectable, and have previously been used as a model of *PRNP* regulation by ER stress. For these reasons, we focused our attention on MCF-7 cells. To determine whether ER stress led to Luman proteolytic cleavage of the N-terminal region, western blot was performed on MCF-7, neurons and astrocytes treated with the ER stressing drugs BFA, Th or TM. The ΔLuman N-terminal region was increased only in cells treated with BFA. A parallel assessment of the chaperone BiP levels confirmed the induction of ER stress by BFA, Th and TM. β-Actin levels were unchanged by ER stress treatments and controlled for equal protein loading (Fig. 1e). The purity of neuronal and astrocytic cultures was assessed by RT-PCR amplification of microtubule-associated protein 2 (*MAP2*) and glial fibrillary acidic protein (*GFAP*) mRNA (Fig. 1f). *MAP2* mRNA was abundant in neuronal, but not astrocytic cultures. Conversely, *GFAP* levels were substantially superior in the astrocytic, than in the neuronal cultures. Neither *MAP2* nor *GFAP* was detected in MCF-7 cells. *HPRT1* controlled for overall mRNA levels. Luman cleavage was further investigated using a Luman construct bearing an N-terminal HA-tag. Once again, BFA treatment, but not Th or TM, led to the cleavage of Luman, as expected^34^ (Fig. 1g), starting as early as thirty minutes after treatment with BFA (Fig. 1h). Taken together, these results show that the ER stressing drug BFA (1) up-regulates *LUMAN* mRNA levels, and (2) promotes Luman activation by cleavage of its N-terminal cytosolic region.

### Luman contributes to BFA-induced PRNP expression

**Figure 2:**
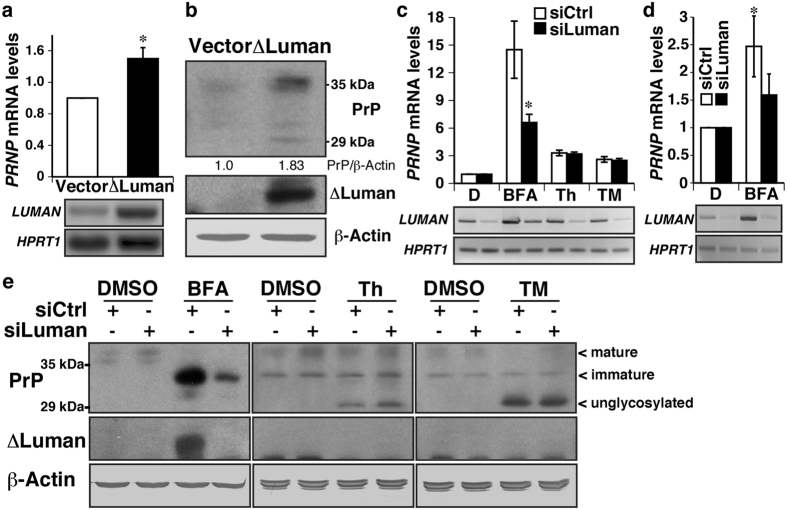
Luman contributes to BFA-induced *PRNP* expression. (**a**) Relative increase in *PRNP* mRNA levels assessed by qPCR 24 h after ΔLuman transfection in MCF-7 cells. Figure represents the mean ± SEM of five experiments, analysed using a unilateral student t-test *p = 0.01. (**b**) Western blot for PrP, ΔLuman and β-Actin of MCF-7 cells transiently transfected with ΔLuman. Relative PrP/β-Actin ratio is indicated, and represents the mean of nine experiments. (**c**) Relative increase in *PRNP* mRNA levels assessed by qPCR in MCF-7 cells transfected with scrambled (siCtrl) or Luman-targeting (siLuman) siRNA, and treated with DMSO (D, 0.1%) or 5 μg/mL brefeldin A (BFA), thapsigargin (Th) or tunicamycin (TM) for 18 h. Data represent the mean ± SEM of seven experiments, analysed using a two-way ANOVA followed by a Bonferroni post-hoc test *p < 0.0001 compared to siCtrl. (**d**) Relative increase in *PRNP* mRNA levels assessed by qPCR in primary human neurons transfected with non-targeting (white) or Luman-targeting (black) siRNA and treated with DMSO (D, 0.1%) or BFA (5 μg/mL), for 18 h. Represents the mean ± SEM of five experiments, analysed using a two-way ANOVA followed by a Bonferroni post-hoc test *p < 0.05 compared to DMSO. **Lower panels** *LUMAN* and *HPRT1* amplicons obtained by RT-PCR. (**e**) Western blot for PrP, ΔLuman and β-Actin of MCF-7 cells transfected for 24 h with scrambled (siCtrl) or Luman-targeting (siLuman) siRNA and treated with DMSO (0.1%) or 5 μg/mL of BFA, Th or TM for 18 h. Mature, immature and unglycosylated PrP bands are indicated. Full-length images of blots and gels are presented in Supplementary Information.

To investigate the role of ΔLuman on *PRNP* expression, we transiently transfected ΔLuman (amino acids 1–215) in MCF-7 cells. Over-expression of ΔLuman led to a 1.5-fold increase in *PRNP* mRNA levels relative to those of empty vector-transfected cells (Fig. 2a). To assess whether this increase in *PRNP* mRNA translated into higher protein levels, PrP levels were assessed by western blot. There was a 1.8-fold increase in PrP levels in ΔLuman-transfected MCF-7 (Fig. 2b). ΔLuman over-expression was confirmed by western blot and β-Actin served as loading control. The increase in PrP-coding mRNA and protein levels suggest that ΔLuman contributes to the expression of the *PRNP* during ER stress. *LUMAN* expression was silenced prior to inducing ER stress to assess its contribution to ER stress-induced *PRNP* expression. In MCF-7 cells, BFA, but not Th or TM, treatment significantly increased *PRNP* mRNA levels when compared to DMSO-treated condition, and Luman silencing attenuated the induction of *PRNP* mRNA by BFA (Fig. 2c). Luman silencing also attenuated BFA-induced *PRNP* mRNA levels in primary human neurons (Fig. 2d). At the protein level, BFA, Th and TM treatments increased PrP levels, compared to DMSO control, in both scrambled and Luman-targeting siRNA transfected cells (Fig. 2e). BFA-induced PrP was immature glycosylated. Th increased unglycosylated PrP and maintained immature and mature glycosylated PrP levels, while PrP was entirely unglycosylated following TM treatment, as expected since TM inhibits N-linked glycosylation of newly synthesized proteins. However, silencing of Luman attenuated BFA-, but not Th- or TM-, induced PrP levels, thereby confirming the contribution of Luman to the regulation of *PRNP* expression during BFA-induced ER stress.

### ΔLuman up-regulates PRNP promoter activity via the ERSE26 element

**Figure 3:**
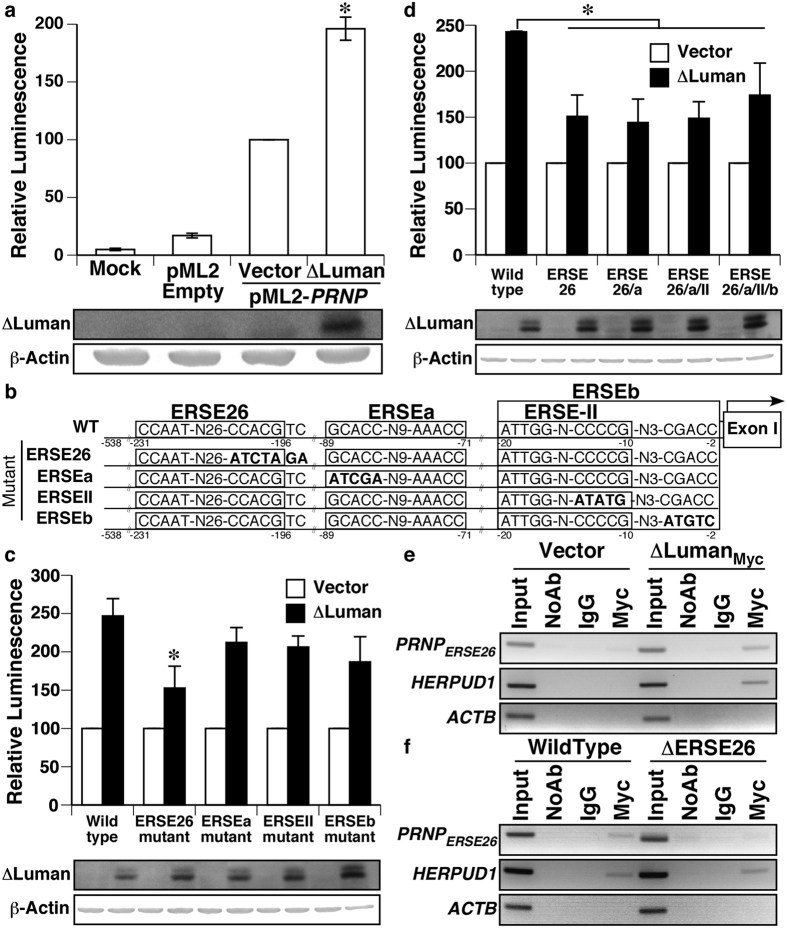
ΔLuman up-regulates *PRNP* promoter activity *via* the ERSE26 element. **(a)** Relative secreted luciferase activity from HEK293T co-transfected with pML2-*PRNP538* and pBud-eGFP or pBud-eGFP-ΔLuman. (**b**) Schematic representation of the wild type and mutated *PRNP* promoter ERSE sites investigated. (**c**) Percentage of increase caused by ΔLuman-Myc over-expression on wild type and mutated *PRNP* promoter luciferase activity. (**d**) Percentage of increase caused by ΔLuman-Myc on wild type and cumulatively mutated *PRNP* promoter luciferase activity. (**a,c,d**) All luciferase experiments represent the mean ± SEM of three independent experiments, analysed using a one-way ANOVA followed by a Dunnett post-hoc test *p < 0.05. **Lower panels** Western blot for ΔLuman and β-Actin. (**e**,**f**) Chromatin immunoprecipitation of HEK293T cells transfected with pBud-eGFP or pBud-eGFP-ΔLuman-Myc (**e**) or wild type or ERSE26 mutant (ΔERSE26) HEK293T cells transfected with pBud-eGFP-ΔLuman-Myc (**f**). PCR amplification of the ERSE26 promoter region, the *HERPUD1* or *ACTB* gene promoters from input or DNA immunoprecipitated with no antibody, non-specific IgG or anti-Myc tag antibody. Full-length images of blots and gels are presented in Supplementary Information.

To determine if ΔLuman up-regulates *PRNP* promoter activity, ΔLuman was co-transfected with the secreted luciferase *PRNP* promoter reporter construct pML2-*PRNP*538 in HEK293T cells to maximize plasmid transfection efficiency. The over-expression of ΔLuman significantly increased *PRNP* promoter activity compared to control (Fig. 3a). Empty vector- and mock-transfected cells showed low luciferase activity. To identify the binding site of ΔLuman to the *PRNP* promoter, we assessed the impact of mutating the ERSE elements of the *PRNP* promoter on the ability of ΔLuman to increase *PRNP* promoter activity (Fig. 3b). Site-directed mutagenesis of the ERSE26 site, but not of the other ERSE elements, reduced by forty percent ΔLuman-induced increase in *PRNP*538 promoter activity (Fig. 3c). Furthermore, cumulative mutations of all the other ERSE elements of the *PRNP* promoter did not further decrease ΔLuman-induced promoter activity compared to the ERSE26 mutant (Fig. 3d). Levels of ∆Luman were verified by western blot analyses in all transfected cells (Fig. 3a,c,d). The results show that, of the four sites investigated, ERSE26 is the only one required for full induction of *PRNP* promoter activity by ΔLuman. Chromatin immunoprecipitation was performed to determine whether ΔLuman interacted with the ERSE26 region of the *PRNP* promoter. To circumvent the lack of highly specific, commercially available antibody against ΔLuman, a Myc-tagged ΔLuman was transiently transfected and immunoprecipitated using an anti-Myc tag antibody. The positive control *HERPUD1*, a known target of Luman, and the *PRNP* ERSE26 promoter region were both amplified from the chromatin of ΔLuman-Myc-transfected cells immunoprecipitated with the anti-Myc antibody, but not from the chromatin of empty vector-transfected cells or samples immunoprecipitated without antibody or with a non-specific IgG. The *ACTB* promoter was not amplified and served as a negative control for ΔLuman–Myc binding (Fig. 3e). Furthermore, *PRNP* ERSE26, but not *HERPUD1*, amplification was lost in genetically altered HEK293T cells lacking the base pairs −202 to −191 of the *PRNP* promoter region (ΔERSE26: 5′-AGCCACGTCAGG-3′), a region that spans the 3′ conserved arm of the ERSE26 element (underlined) (Fig. 3f).

### Luman and XBP1 both contribute to BFA-induced PRNP expression

**Figure 4:**
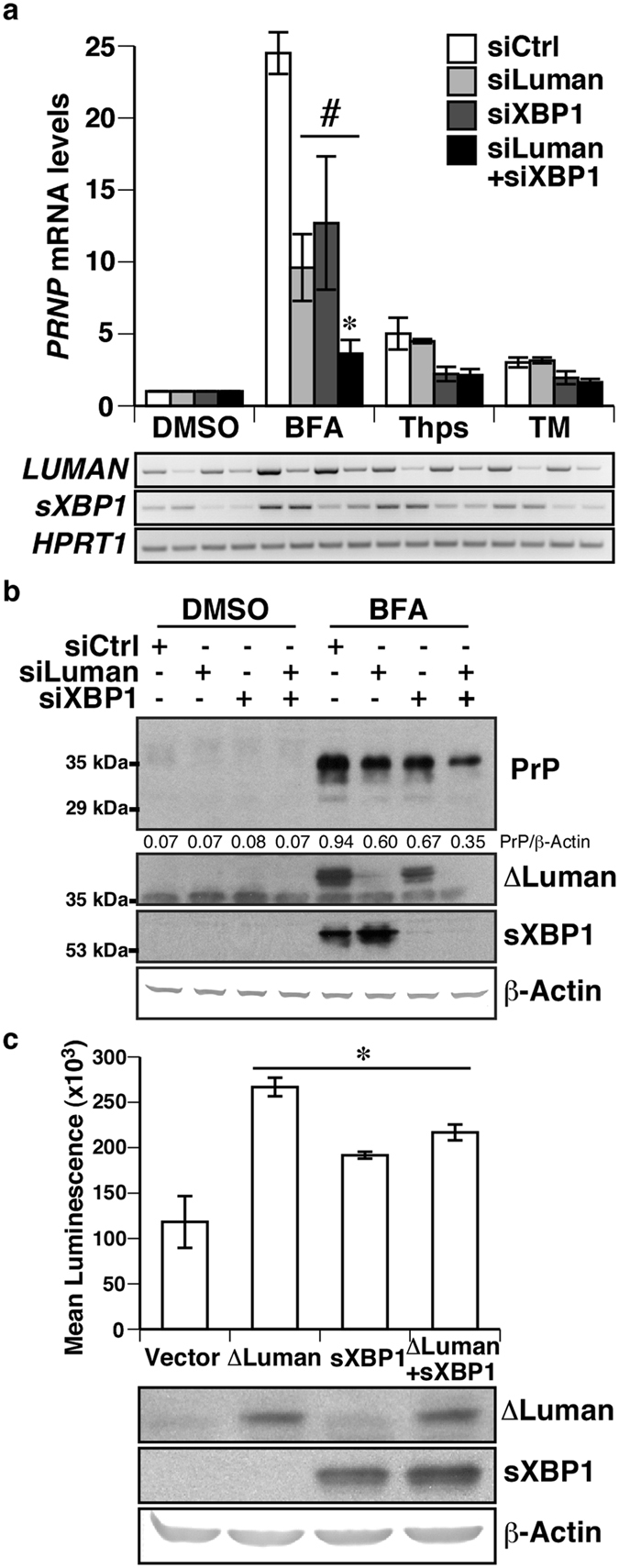
Luman and sXBP1 both contribute to BFA-induced *PRNP* expression. **(a)** Relative increase in *PRNP* mRNA levels assessed by qPCR in MCF-7 cells transfected with scrambled (siCtrl), Luman- (siLuman) or XBP1-targeting (siXBP1) siRNA, and treated with DMSO (0.1%) or 5 μg/mL of brefeldin A (BFA), thapsigargin (Thps) or tunicamycin (TM) for 18 h. Represents the mean ± SEM of three duplicated experiments, analysed using a two-way ANOVA followed by a Bonferroni post-hoc test #*p* < 0.05 compared to siCtrl, *p < 0.05 compared to siLuman or siXBP1. **Lower panel** *LUMAN*, s*XBP1* and *HPRT1* amplicons obtained by RT-PCR. (**b**) Western blot for PrP, XBP1, Luman and β-Actin of MCF-7 cells transfected for 24 h with scrambled (siCtrl), Luman- or XBP1-targeting siRNA and treated with either DMSO or BFA (5 μg/mL) for 18 h. PrP/β-Actin ratio is indicated, and represents the mean of three experiments. (**c**) *PRNP* promoter luciferase activity following transfection with ΔLuman-Myc, spliced XBP1 (sXBP1), or ∆Luman-Myc and sXBP1 in HEK293T cells. Data represent the mean ± SEM of three independent experiments, analysed using a one-way ANOVA followed by a Dunnett post-hoc test *p < 0.05 compared to DMSO. **Lower panel** Western blot for ΔLuman-Myc, sXBP1 and β-Actin. Full-length images of blots and gels are presented in Supplementary Information.

To assess whether silencing both Luman and XBP1 would be sufficient to fully suppress ER stress-induced *PRNP* expression, one or both transcription factors were silenced prior to treating MCF-7 cells with ER stressing drugs. BFA treatment led to an increase in both *PRNP* mRNA and protein levels, and silencing of either Luman or XBP1 attenuated by thirty-five percent BFA-induced increase in *PRNP* expression. Furthermore, silencing of both Luman and XBP1 further reduced ER stress-induced *PRNP* mRNA and protein levels (Fig. 4a,b). Induction and silencing of ΔLuman and sXBP1 protein levels was confirmed by RT-PCR or western blot. To determine whether ΔLuman and sXBP1 synergistically regulate *PRNP* promoter activity, Luman and/or sXBP1 were co-transfected with the pML2-*PRNP538* reporter plasmid. Both ΔLuman and sXBP1 increased *PRNP* promoter activity when compared to empty-vector control (Fig. 4c). However, over-expression of both ΔLuman and sXBP1 did not further increase *PRNP* promoter activity. Over-expression of ΔLuman and sXBP1 was confirmed by western blot.

### PrP does not influence the degradation of ERAD substrate Transthyretin D18G (TTR^D18G^)

**Figure 5:**
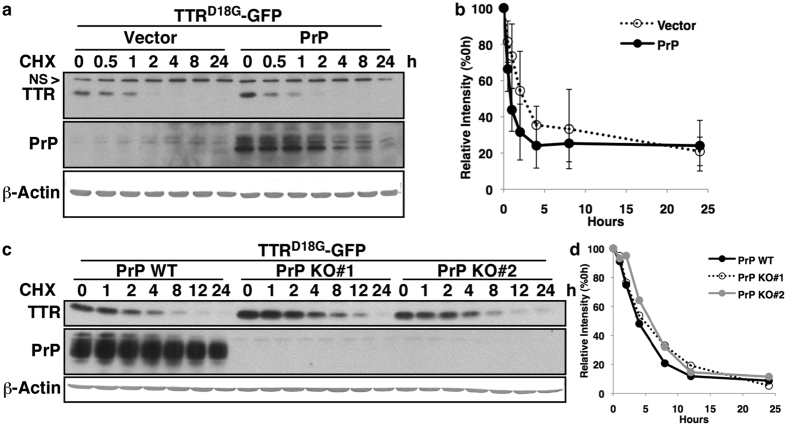
PrP does not influence the degradation of ERAD substrate Transthyretin^D18G^ (TTR^D18G^). **(a)** Western blot for TTR^D18G^-eGFP, PrP and β-Actin of MCF-7 cells co-transfected with pBud-eGFP (Vector) or pBud-eGFP-PrP (PrP) and pcDNA3.1(−)-TTR^D18G^-GFP, following a cycloheximide chase (75 μg/mL), with antibodies against eGFP (B-2), PrP (3F4) and β-Actin (AC15). (**b**) Schematic representation of quantified TTR^D18G^-GFP levels shown in (**a**) Data represent the mean ± SEM of three independent experiments. (**c**) Western blot for TTR^D18G^-eGFP, PrP and β-Actin of wild type or the PrP KO#1 and KO#2 CR7 cell lines transfected with pcDNA3.1(−)-TTR^D18G^-GFP, following a cycloheximide chase (75 μg/mL). (**d**) Schematic representation of quantified TTR^D18G^-GFP levels shown in (**c**) Data represent the relative intensity of a single experiment involving two independent mutant cell lines. Full-length images of blots are presented in Supplementary Information.

To better understand the physiological relevance of the regulation of *PRNP* expression by Luman and XBP1 during ER stress, and because both XBP1 and Luman regulate several genes involved in ERAD, the impact of PrP over-expression on the degradation rate of the ERAD substrate TTR^D18G^-GFP was assessed. The results show that PrP over-expression in the low PrP-expressing MCF-7 cell line did not facilitate TTR^D18G^-GFP degradation during a cycloheximide chase (Fig. 5a,b). Conversely, the degradation rate of the TTR^D18G^-GFP substrate was assessed in high PrP-expressing CR7 glioblastoma cells that underwent CRISPR/Cas9-mediated PrP knockout (KO) (Fig. 5c). No difference was observed between the degradation rate of TTR^D18G^-GFP in WT and PrP KO CR7 cells (Fig. 5c,d). In summary, these findings argue against a role of PrP in modulating ERAD.

### PrP does not influence epoxomicin-induced Caspase 3/7 DEVDase activity

**Figure 6:**
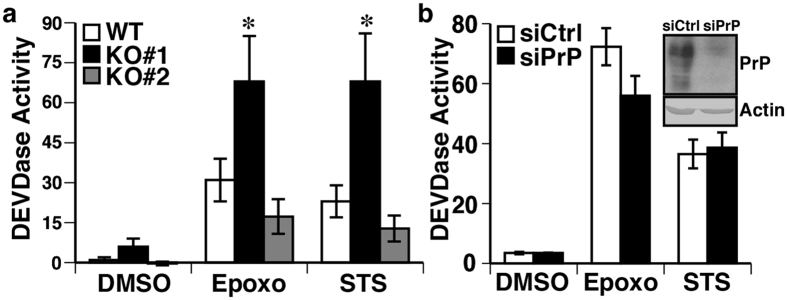
PrP does not influence epoxomicin-induced Caspase 3/7 DEVDase activity. **(a)** DEVDase activity (fmol AFC/min/μg) of wild type and two PrP-disrupted CR7 cell lines treated with DMSO (0.1%), epoxomicin (Epoxo, 0.5 μM) or staurosporine (STS, 0.25 μM) for 18 h. Data represent the mean ± SEM of five independent experiments, analysed using a two-way ANOVA followed by a Dunnett post-hoc test *p < 0.05 compared to DMSO. (**b**) DEVDase activity (fmol AFC/min/μg) of CR7 cells transfected with scrambled (siCtrl) or PrP-targeting (siPrP) siRNA treated with 0.1% DMSO, 0.5 μM epoxomicin (Epoxo) or 0.25 μM staurosporine (STS) for 18 h. Data represents the mean ± SEM of three independent experiments, analysed using a two-way ANOVA. **Inset** Western blot for PrP of CR7 transfected with scrambled (siCtrl) or PrP-targeting (siPrP) siRNA. Full-length images of blots are presented in Supplementary Information.

Because (1) proteasomal inhibition causes ER stress and apoptosis, (2) Luman protects against ER stress-induced Caspase-3 activity^36^, and (3) PrP protects against ER stress-induced cell death^10^, the possibility that Luman–mediated *PRNP* expression protects against proteasomal inhibition-induced cell death was investigated. The impact of PrP on proteasomal inhibition-induced apoptosis was assessed by measuring Caspase-3/7 DEVDase activity in the WT or the CRISPR/Cas9-generated PrP KO CR7 cell lines (KO#1, KO#2) treated with epoxomicin. Cells treated with staurosporine served as positive control. Epoxomicin and staurosporine caused a twenty-five- to thirty-fold increase in DEVDase activity. Strangely, epoxomicin- and staurosporine-induced DEVDase activity was exacerbated in the PrP KO#1 cell line, but not in the PrP KO#2 cell line (Fig. 6a). To determine which effect is due to the loss of PrP, siRNA-mediated knockdown of PrP was achieved in CR7. Silencing of PrP expression did not significantly influence epoxomicin- or staurosporine-induced DEVDase activity (Fig. 6b). These results suggest that CRISPR-targeted knockdown has non-target effects in the CR7 KO#1 cell line, and that PrP does not protect CR7 cells against proteasomal inhibition-induced apoptosis.

### Atorvastatin-induced neuritogenesis is independent of Luman activity

**Figure 7:**
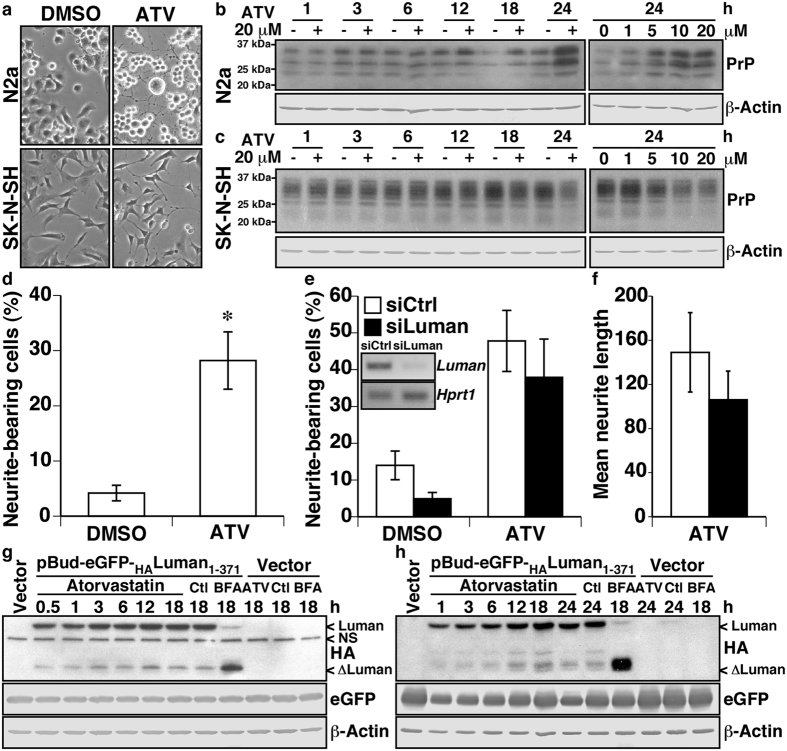
Atorvastatin-induced neuritogenesis is independent of Luman activity. **(a)** Representative image of neuritogenesis in N2a and SK-N-SH neuroblastoma cells following atorvastatin (ATV, 20 μM) treatment for 24 h. (**b**,**c**) Western blot for PrP and β-Actin of N2a (**b**) or SK-N-SH (**c**) cells treated with 20 μM atorvastatin (ATV) for increasing amounts of time (0–24 hrs) or drug concentrations (0–20 μM). (**d**) Quantification of neurite-bearing N2a cells following treatment with 0.1% DMSO or 20 μM ATV for 24 h. Data represent the average percentage of neurite-bearing cells (mean ± SEM, *n* = 10 images), analysed using a unilateral student t-test *p < 0.001. (**e**) Quantification of neurite-bearing N2a cells transfected with scramble (siCtrl) or Luman-targeting siRNA (siLuman), following 0.1% DMSO or 20 μM ATV for 24 h. **Inset** *Luman* and *Hprt1* amplicons obtained by RT-PCR from siCtrl- or siLuman-transfected N2a cells. Data represent the average percentage of neurite-bearing cells (mean ± SEM, *n* = 10 images), analysed using a two-way ANOVA. (**f**) Quantification of mean neurite length of N2a cells transfected with scramble (white) or Luman-targeting siRNA (black), following 20 μM ATV for 24 h. Data represent the average of total neuritic length (pixel)/total number of cells (mean ± SEM, *n* = 10 images), analysed using a bilateral student t-test. (**g**,**h**) Western blot for the Luman N-terminal HA tag, eGFP or β-Actin of N2a (**g**) or HEK293T (**h**) cells transfected with pBud-eGFP (Vector) or an N-terminal HA-tagged full length Luman construct (pBud-eGFP-_HA_Luman_1-317_), and treated with 0.1% DMSO (Ctl), 5 μg/mL brefeldin A (BFA) or 20 μM ATV, for increasing amounts of times (0–18 hrs). NS designates an unspecific band. Full-length images of blots are presented in Supplementary Information.

Given that (1) Luman was recently shown to promote axonal growth^40^, (2) Luman regulates several genes involved in cholesterol metabolism^38^, and (3) the cholesterol synthesis inhibitor atorvastatin was reported to promote neuritogenesis in N2a cells by increasing PrP levels^41^, the contribution of Luman to atorvastatin-induced neuritogenesis was investigated. As expected, a 20 μM atorvastatin treatment increased neuritogenesis in N2a and, to a lesser extent, the human neuroblastoma SK-N-SH cell line after 24 h (Fig. 7a). Atorvastatin increased PrP in a dose- and time-dependent manner in N2a cells (Fig. 7b), but had the opposite effect in the SK-N-SH cell line (Fig. 7c). Quantification of neurite-bearing N2a cells confirmed neurite formation following atorvastatin treatment (Fig. 7d). However, despite a trend towards lower amounts of atorvastatin-induced neurite-bearing (Fig. 7e), and mean neuritic length (Fig. 7f) in N2a cells, no statistically significant effect was observed by siRNA-targeted knock down of Luman. Furthermore, while Luman proteolytic activation was clearly increased by BFA treatment, the levels of ∆Luman were similar in atorvastatin- and DMSO- treated N2a (Fig. 7g) and HEK293T (Fig. 7h) cells, thus indicating that atorvastatin does not potently induce Luman activation. Blotting for β-Actin and eGFP controlled for equal loading and transfection efficiency, respectively. Overall, these data confirm previous reports that atorvastatin increases neuritogenesis and PrP levels in N2a cells. In addition, the results indicate that Luman is not necessary for atorvastatin-induced neuritogenesis, and exclude atorvastatin as a regulator of Luman proteolytic activation in murine and human cells. Contrary to N2a cells, atorvastatin reduces PrP levels in human SK-N-SH neuroblastoma cells.

## Discussion

The results of this study show that the ER stressing drug, BFA, up-regulates Luman activity in several human cell types, and that Luman contributes to BFA-induced *PRNP* expression by interacting with the ERSE26 of the *PRNP* promoter. Attempts to understand the function of Luman-induced *PRNP* expression excluded a role of PrP in promoting ERAD, protecting against epoxomicin-induced apoptosis, and atorvastatin-induced neuritogenesis.

Luman activation was observed both by increased mRNA levels and proteolysis into ∆Luman by BFA-, but not TM- or Th-, treatment. The ability of BFA to increase *LUMAN* mRNA levels by five- to ten-fold in human CNS neurons and astrocytes and breast carcinoma MCF-7 cells, has not been previously reported. In contrast, Th, TM and the proteasomal inhibitor, MG132, up-regulates mRNA levels by three- to six-fold *LUMAN* in HEK293 and C6 glial cells^36^,^42^. Our results are consistent with previous reports that BFA, but not Th and TM, triggers Luman proteolytic activation in HEK293, Vero, RAW264 and dendritic cells^34^,^37^,^43^,^44^. Together, these results identify BFA as a most potent ER stressing inducer of *LUMAN* transcription in several cell types, and suggest that the induction of *LUMAN* transcription by Th and TM may be cell type specific.

The presence of *LUMAN* mRNA in MCF-7, neurons and astrocytes, and its responsiveness to BFA made Luman the most relevant OASIS family member to study as a mediator ER stress-induced *PRNP* expression. Our results identify Luman as a new regulator of *PRNP* expression. Over-expression of ΔLuman led to a 1.5-fold increase in *PRNP* mRNA and a 2.0- to 2.5-fold increase in *PRNP* promoter activity. This is comparable to the regulation of *PRNP* expression by sXBP1, which leads to a 2.0-fold increase in *PRNP* mRNA when over-expressed in MCF-7 cells^30^, indicating that Luman is as potent as the canonical UPR mediator sXBP1 at promoting *PRNP* expression. The level of *PRNP* induction was similar to the 2.0-fold induction in *EDEM* mRNA caused by ΔLuman in HEK293 cells^37^, but was much lower than the 9.0-fold increase of *HERPUD1* mRNA^36^ and 4.5-fold induction of *HERPUD1* promoter activity^36^. Moreover, silencing of Luman substantially attenuated BFA-induced *PRNP* expression, an effect previously observed in XBP1-silenced MCF-7 cells^30^, and co-silencing Luman and XBP1 led to a greater reduction in BFA-induced *PRNP* expression than silencing each individually. However, the inability of Luman and XBP1 co-silencing to completely abrogate BFA-induced *PRNP* implies that other factors are implicated in this regulation.

The discovery that Luman regulates *PRNP* expression brings us closer to achieving modulation of ER stress-induced PrP levels, by providing a novel target for pharmacological intervention. Because (1) PrP levels influence the progression of prion diseases^1^,^2^,^3^, (2) protects against apoptosis in a broad range of cancers^45^,^46^,^47^, and (3) ER stress is detected in both prion disease^48^ and solid tumors^49^,^50^, pharmacological attenuation of ER stress-induced *PRNP* expression could constitute a promising strategy in the treatment of these disorders. The recent discovery that Ceapins can specifically inhibit ATF6α proteolytic activation, a target previously perceived as “undruggable”, suggests that similar inhibitors could also be developed against Luman^51^.

The identification of Luman as a regulator of *PRNP* expression also helps understand the physiological purpose of PrP. In this study, we excluded three potential functional implications of Luman-regulated *PRNP* expression: promoting ERAD, protecting against proteasomal inhibition-induced cell death, and mediating atorvastatin-induced neuritogenesis. Although Luman regulates the expression of ERAD-related *EDEM*^37^, *HERPUD1*^36^, *Canx* and *Ubxn4*^38^, neither CRISPR/Cas9-mediated disruption, nor over-expression of PrP influenced the degradation rate of the ERAD substrate TTR^D18G^. Our data also show that neither CRISPR/Cas9-mediated disruption, nor siRNA-silencing of *PRNP* expression significantly altered epoxomicin- or staurosporine-induced Caspase 3/7 DEVDase activity. This contrasts with the ability of PrP to protect against ER stress-induced cell death^10^, but confirms previous results from our lab showing that PrP does not attenuate staurosporine-induced cell death^15^, and signifies that the ability of *PRNP* to protect against cell death is not universal, and may be influenced by the nature of the cell death stimulus. From a therapeutic standpoint, the inability of PrP to protect against proteasomal inhibition-induced Caspase 3/7 DEVDase activity implies that up-regulation of *PRNP* expression would not contribute to chemoresistance in cancers treated with proteasomal inhibitor bortezomib, such as multiple myeloma or mantle cell lymphoma. Lastly, this study investigated whether atorvastatin stimulates neuritogenesis through Luman-mediated *PRNP* expression. Our data confirm that atorvastatin induces neuritogenesis, and increases PrP levels in N2a neuroblastoma^41^. However, siRNA-mediated silencing of Luman does not significantly reduce atorvastatin-induced neuritogenesis, nor does atorvastatin trigger Luman activation, thereby excluding Luman induced *PRNP* expression as a mediator of atorvastatin-induced neuritogenesis. The exact function of Luman-mediated up-regulation of *PRNP* gene expression thus remains to be discovered remains a difficult task in the absence of a clear function for Luman.

Overall, this study describes an unprecedented induction of *LUMAN* transcription by BFA that reflects a role in long-term cell adaptation, and refines our understanding of UPR-mediated *PRNP* expression by identifying Luman as a novel mediator of BFA-induced *PRNP* expression. This regulation offers a new pharmacological target to attenuate *PRNP* expression, and designates PrP as an effector of Luman function. Although, this function remains unclear, current results exclude the regulation of *PRNP* expression by Luman as a mean to facilitate ERAD, protect against proteasomal inhibition-induced apoptosis or promote atorvastatin-induced neuritogenesis.

## Methods

### Cell culture and pharmacological treatments

HEK293T, N2a, and SK-N-SH cells (from ATCC, Manassas VA) were cultured in DMEM, MCF-7 in RPMI1640. CR7 cells, which express high endogenous levels of PrP, were derived from a human glioblastoma, cultured in OptiMEM (Gibco Life Technologies, NY), and obtained from Dr Melinda Estes (Cleveland Clinic, Cleveland, OH)^52^. All culture media were supplemented with 10% fetal bovine serum. Neurons and astrocytes were prepared as previously described^53^, with the ethical approval of the McGill University Institutional Review Board. Pharmacological induction of ER stress was achieved using BFA, thapsigargin (Th) or tunicamycin (TM) at a final concentration of 5 μg/mL for all three drugs, doses that maximize ER stress-induced PrP gene expression without significant toxicity to the cells, as described previously^15^. Unless specified, atorvastatin, epoxomicin, staurosporine and cycloheximide were used at a final concentration of 20 μM, 0.5 μM, 0.25 μM and 75 μg/mL, respectively. All drugs were dissolved in dimethyl sulfoxide (DMSO). For all experiments, the final DMSO concentration did not exceed 0.1%.

### RNA purification, reverse transcription and polymerase chain reaction (PCR)

Total RNA was purified using Trizol (Invitrogen, Carlsbad CA) and reverse transcription was performed on 1 μg of total RNA using AMV-RT, poly-dT, RNAase inhibitor and the following protocol: 10 min at 25 °C, 60 min at 42 °C, 5 min at 99 °C, and 5 min at 4 °C. The resulting cDNA was used as template for PCR amplification. The sequences of the primers used for PCR amplification are listed in Table 1. The PCR protocol for all OASIS family members and mouse *Hprt1* was 1 cycle of 5 min at 95 °C, 35 cycles of 30 sec at 95 °C, 1 min at 58 °C, and 30 sec at 68 °C, followed by 1 cycle of 1 min at 68 °C. Identical conditions were used for human *HPRT1* amplification, except that an annealing temperature of 62 °C was used instead of 58 °C. *PRNP* was amplified using 1 cycle of 5 min at 95 °C, 35 cycles of 30 sec at 95 °C, 1 min at 60 °C, and 2 min at 72 °C, followed by 1 cycle of 4 min at 72 °C. Amplification of *MAP2* was achieved using 1 cycle of 5 min at 95 °C, 40 cycles of 30 sec at 95 °C, 1 min at 61 °C, and 1 min at 68 °C, followed by 1 cycle of 2 min at 68 °C. A similar protocol was used for *GFAP*, but required 25 cycles and an annealing temperature of 66.1 °C. A protocol of 1 cycle of 5 min at 95 °C, 30 cycles of 30 sec at 95 °C, 1 min at 60 °C, and 30 sec at 68 °C, followed by 1 cycle of 1 min at 68 °C was used to amplify s*XBP1*.

### Quantitative PCR (qPCR)

qPCR was performed using SYBR Green Taq Mastermix (Quanta Biosciences, Gaithersburg MD) on an Applied Biosystems 7500Fast qPCR apparatus (Invitrogen, Carlsbad CA). Output data was expressed as fold-induction over control condition following normalization to *HPRT1*, using Pfaffl’s method^54^. The qPCR primer sequences used for *PRNP, LUMAN, HSPA5*, and *HPRT1* are listed in Table 1.

### Transfection of plasmids and siRNAs

Transfection of MCF-7 or CR7 cells with either plasmid DNA or siRNA was achieved by nucleofection (Nucleofection kit V, VCA-1003, Lonza, Basel Switzerland). Briefly, 4 million cells were resuspended in nucleofection buffer with either 2 μg or 4 μg plasmid DNA or 300 nM siRNA before nucleofecting (protocol P-020). Cells were then plated at 1 million cells per well and incubated 24 h before use. Lipofectamine RNAiMAX (Invitrogen, Carlsbad CA) was used for siRNA transfection in N2a cells (37.5 pmol/50,000 cells) 24 h before treatment. Single scrambled (sc-37007) sequence and pools of three siRNAs targeting PrP (sc-36318), Luman (sc-37702), murine Luman (sc-37703) or XBP1 (sc-38627) were purchase from Santa Cruz Biotechnologies (Dallas, TX). Silencing of Luman in primary human neurons was performed using Accell SMARTpool scrambled (D-001910-10-20) or Luman-targeting (E-017471-00) pool of four siRNAs (Dharmacon, Lafayette, CO).

### Western blot

Cells were lysed in lysis buffer [50 mM Tris-HCl pH 8.0, 150 mM NaCl, 1% NP-40 and 0.5 mM ethylenediaminetetraacetic acid (EDTA) pH 8.0, 38 μg/mL 4-(2-aminoethyl) benzenesulfonyl fluoride (AEBSF), 0.5 μg/mL leupeptin, 0.1 μg/mL pepstatin, 0.1 μg/mL N-α-p-tosyl-L-lysinechloromethyl ketone hydrochloride (TLCK)]. Samples were incubated on ice before centrifugation at 15,000 g for 10 min. Supernatants were collected and quantified using the bicinchoninic acid method, following the manufacturer’s guidelines (Thermo Scientific, Waltham MA). Equal amounts of protein were diluted in loading buffer (2% SDS, 5% β-mercaptoethanol, 10% glycerol, 0.01% bromophenol blue, 62.5 mM Tris-HCl, pH 6.8) and boiled 5 min before loading on 10 or 15% poly-acrylamide gels. Following electrophoresis, proteins were transferred to polyvinylidene fluoride membranes using a Turbo blot apparatus (BioRad, Hercules CA). Membranes were blocked in 5% milk for one hour before probing with anti-Luman (1:200, M13, kindly provided by Dr. Vikram Misra, University of Saskatchewan, Saskatoon, SK), anti-PrP (1:10,000, 3F4, PrP_109-112_ or 1:500 ab52604, PrP_214-230_, Abcam, Toronto, ON), anti-BiP (C50B12, 1:5,000, Cell Signaling, Beverly MA or H-129 (1:2,500, Santa-Cruz Biotechnology, Dallas, TX), anti-HA (1:2,500, 16B12, Covance, Princeton NJ), anti-XBP1 (M-186 1:200, Santa Cruz Biotechnology, Dallas, TX), anti-GFP (B-2 1:2,500 Santa Cruz Biotechnology, Dallas, TX), anti-Bim (Y-36 1:5,000, Epitomics, Burlingame, CA, USA) or anti-β-Actin (1:5,000, AC15, Sigma, St-Louis MO) antibodies. Detection of primary antibodies was achieved using an HRP-coupled anti-mouse (1:5,000, NA9310, GE Healthcare, Baie-D’Urfe QC) or anti-rabbit (1:5,000, P0217, Dako, Burlington ON) secondary antibody, chemiluminescent substrate (RPN2232, GE Healthcare, Baie-D’Urfe QC) and Kodak BioFilms (Kodak, San Diego CA). β-Actin was revealed using an alkaline phosphatase-coupled anti-mouse antibody (1:5,000, 115-055-003, Jackson ImmunoResearch, West Grove, PA) and the nitro blue tetrazolium/5-bromo-4-chloro-3-indolyl-phosphate substrate.

### Cloning of HA-Luman, ΔLuman and ΔLuman-Myc

The DNA sequence coding for HA-Luman was amplified from MCF-7 cDNA with high fidelity Pfu polymerase (Agilent Technologies, Santa Clara CA) using the forward 5′-CCG CTA AAG CTT ACC ATG GCA TAC CCA TAC GAC GTC CCA GAC TAC GCT GAG CTG GAA TTG GAT GCT GGT GA-3′ and the reverse 5′-ACG CGA GTC GAC TAG CCT GAG TAT CTG TCC TGC-3′ primers, while amplification of the sequence coding for Luman amino acid 1 to 215 was accomplished using the forward 5′-CCG CTA AAG CTT AGC ATG GAG CTG GAA TTG GAT GC-3′ and reverse 5′-ACG CGA GTC GAC TAG GCC TGG AGT TTC CTC AGT TG-3′ primers. Both pairs introduced flanking HindIII and SalI sites used for cloning into the pBud-eGFP vector^55^. pBud-eGFP-ΔLuman-Myc was generated by mutating the stop codon by QuikChange Site-Directed Mutagenesis (Stratagene, LaJolla, CA), using the forward primer 5′-CTG AGG AAA CTC CAG GCC TCG TCG ACA TCG ATC TTA AGC-3′ and its reverse complement. This allowed the translation of a C-terminal Myc-tag already present in the pBud-eGFP vector.

### Cloning of pML2-PRNP538 and mutagenesis of ERSE sites

The *PRNP* promoter regions −538: +125 was obtained from the pGL2-*PRNP*538 plasmids, kindly provided by Dr. John Collinge (MCR Prion Unit, London). Briefly, the promoter region was excised using BglII and ligated into the secreted luciferase reporter pML2 plasmid (Clontech, Mountain View CA) between the BglII and HindIII sites. Site-directed mutagenesis of the pML2-*PRNP*538 plasmid was accomplished by QuikChange Site-Directed Mutagenesis (Stratagene, LaJolla, CA) using the following forward primers and their reverse complements: ERSE-like 5′-AAG ATG ATT TTT ACA GTC AAT GAG ATC TAG AAG GGA GCG ATG GCA CCC GCA GG-3′, in ERSEa 5′-CGG CCC TGC TTG GCA GCG CGA TCG ACT TTA ACT TAA ACC TCG GC-3′, in ERSE-II 5′-GCG CGG CAA TTG GTC ATA TGG CCG ACC TCC GCC CGC G- 3′, and in ERSEb 5′-GCG GCA ATT GGT CCC CGC ATA TGT CTC CGC CCG CGA GCG CCG-3′. To mutate the ERSEb site next to the already mutated ERSEII site the following forward primer and its reverse complement were used: 5′-GCG GCA ATT GGT CAT ATG ATA TGT CTC CGC CCG CGA GCG CCG-3′.

### Luciferase activity assay

HEK293T were used because they are highly transfectable. Cells were seeded at 300,000 cells/well in 6-well plates and incubated overnight. Transfection was performed using the polyethylenimine (PEI, Polyscience Inc., Warrington PA) method^56^. Briefly, 2 μg of pML2-Luciferase reporter DNA and 2 μg of pBud-eGFP or pBud-eGFP-ΔLuman were co-diluted in OptiMEM (Invitrogen, Carlsbad CA) and combined to 20 μg of PEI. After 20 min at room temperature, the complex was added to each well in a drop-like manner. Culture media was collected after 24 h, and secreted luciferase activity was assessed in duplicate for each sample using the Ready-to-glow substrate, according to manufacturer’s protocol (Clontech, Mountain View CA), and a H4 plate reader (Biotek, Winooski VT).

### CRISPR/Cas9-mediated mutagenesis

Guide RNAs targeting the protospacer adjacent motifs (PAM) found at the *PRNP* Tyr38 codon or the ERSE26 promoter region were generated by inserting the oligos 5′-ACT GGG GGC AGC CGA TAC C-3′ and 5′-ACA GTC AAT GAG CCA CGT C-3′ in the pX330-U6-CBh-hSpCas9 plasmid, respectively. pX330-U6-Chimeric_BB-CBh-hSpCas9 was a gift from Dr. Feng Zhang (Addgene plasmid # 42230). The resulting plasmids were co-transfected with a GFP-expressing plasmid, and GFP-positive cells were individually plated in 96-well plates using a BD FACS Aria Fusion cell sorter. Screening of clones in which the *PRNP* open reading frame was disrupted was achieved by western blot using the anti-PrP 3F4 antibody. The ERSE26 mutants were screened by restriction digest. Briefly, genomic DNA was purified as previously described^57^, and used as a template for RT-PCR amplification of the *PRNP* −536:-137 promoter region, using the forward 5′-CGG AGC GCA TTT TTC TCA TTT G-3′ and reverse 5′-GAG ATT CGC TTG AAC ACT TG-3′ primers. Amplicons were then digested using the restriction enzyme BmgBI (5′-CAC/GTC-3′). Only wild type amplicons were susceptible to BmgBI digestion. The mutation of selected clones was characterized by Sanger sequencing.

### Chromatin immunoprecipitation

Wild type or ΔERSE26 mutant HEK293T cells were grown to seventy percent confluence in T75 flasks, and transfected with pBud-eGFP or pBud-eGFP-ΔLuman-Myc using the PEI method^56^. After 24 h, cells were recuperated by trypsinization, rinsed with phosphate-buffered saline, and cross-linked for 10 min at room temperature using 1% formaldehyde. The cross-linking reaction was stopped by bringing the solution to 0.125 M glycine, before rinsing, and lysing the cells in swelling buffer (10 mM Tris-HCl pH 8.0, 0.25 M sucrose, 0.5% NP-40, 2 mM dithiothreitol (DTT), 38 μg/mL AEBSF, 0.5 μg/mL leupeptin, 0.1 μg/mL pepstatin and 0.1 μg/mL TLCK) for 10 min on ice. The nuclei were then pelleted and sonicated on ice for 20 min in sonication buffer (50 mM 4-(2-hydroxyethyl)-1-piperazine-ethane-sulfonic acid (HEPES) pH 7.4, 140 mM NaCl, 1 mM EDTA, 1% Triton X-100, 0.1% sodium deoxycholate, 1% SDS, 38 μg/mL AEBSF, 0.5 μg/mL leupeptin, 0.1 μg/mL pepstatin and 0.1 μg/mL TLCK) to shear the DNA. The nuclear lysate was pre-cleared with protein G-sepharose beads coated with 1 μg/mL sonicated salmon sperm nuclei (S3126, Sigma, St-Louis MO) and 1 mg/mL bovine serum albumin. Immunoprecipitation was performed overnight with no antibody, normal serum IgG or anti-Myc tag antibody (1:1,000, 9B11, Cell Signaling, Beverly MA). The beads were washed twice with sonication buffer, wash buffer A (50 mM HEPES pH 7.4, 500 mM NaCl, 1 mM EDTA, 1% Triton X-100, 0.1% sodium deoxycholate, 0.1% SDS, 38 μg/mL AEBSF, 0.5 μg/mL leupeptin, 0.1 μg/mL pepstatin and 0.1 μg/mL TLCK), wash buffer B (20 mM Tris pH 8.0, 1 mM EDTA, 250 mM LiCl, 0.5% NP-40, 0.5% sodium deoxycholate, 38 μg/mL AEBSF, 0.5 μg/mL leupeptin, 0.1 μg/mL pepstatin and 0.1 μg/mL TLCK), Tris-EDTA solution (10 mM Tris-HCl, 1 mM EDTA pH 8.0) and then eluted in elution buffer (50 mM NaHCO_3_, 1% SDS, 1 mM EDTA and 50 mM Tris-HCl pH 8.0). Cross-linking was reversed by bringing the solution to 238 mM NaCl and incubating at 65 °C overnight. Immunoprecipitated DNA fragments were purified using the phenol:chloroform method and used as template for PCR. The primers pairs used to amplify the *PRNP* ERSE26 region, *HERPUD1* and *ACTB* promoter are listed in Table 1.

### DEVDase activity assay

Cells were lysed in lysis buffer (50 mM 4-(2-hydroxyethyl)-1-peperazineethanesulfonic acid, 0.1% 3-[(3-cholamidopropyl)-dimethylammonio]-1-propanesulfonate (CHAPS), 0.1 mM EDTA) and the lysate protein concentration was quantified by the Bradford method^58^. Cell lysates (20–30 μg protein) were combined with Stennicke’s buffer (20 mM piperazine-N,N’-bis (2-ethanesulfonic acid) (PIPES), 30 mM NaCl, 1 mM EDTA, 0.1% CHAPS, 10% sucrose) containing 10 μM N-Acetyl-Asp-Glu-Val-Asp-7-amido-4-trifluoromethylcoumarin (Ac-DEVD-AFC) and 10 mM DTT^59^. The fluorogenic reaction took place at 37 °C, and the fluorescence level (Excitation 380 nm: Emission 505 nm) was acquired every minute for an hour in a black, clear bottomed, 96-well plate, using a H4 plate reader (Biotek, Winooski VT).

### Statistical analysis

The statistical significance between *LUMAN* or *HSPA5* mRNA levels was determined using a one-way ANOVA and a Dunnett post-hoc test (compared to DMSO control). For the *PRNP* mRNA levels following ΔLuman over-expression or the induction of neurite formation and length by atorvastatin, significance was assessed using a unilateral student t-test assuming equal variance. In Luman-silenced cells, the induction of *PRNP* mRNA levels by ER stress and the induction of neuritogenesis by atorvastatin were analysed using a two-way ANOVA and Bonferroni post-hoc tests. Caspase3/7 DEVDase activity of PrP-disrupted and PrP-silenced cells was analysed using a two-way ANOVA and a Dunnett post-hoc test, when applicable. All luciferase activity experiments were analyzed using one-way ANOVAs and Dunnett post-hoc tests. For all experiments, a *p*-value of less than 0.05 was considered significant.

## Additional Information

**How to cite this article:** Déry, M.-A. and LeBlanc, A. C. Luman contributes to brefeldin A-induced prion protein gene expression by interacting with the ERSE26 element. *Sci. Rep.* **7**, 42285; doi: 10.1038/srep42285 (2017).

**Publisher's note:** Springer Nature remains neutral with regard to jurisdictional claims in published maps and institutional affiliations.

## Supplementary Material

Supplementary Information

## Figures and Tables

**Table 1:** Primer sequences used for PCR.

ID		Sequence
*PCR*		
*OASIS*	F	5′-ACCTGGACCACTTTACGGAG-3′
	R	5′-TGGTGTCCTCCATCTTGATG-3′
*BBF2H7*	F	5′-CCTTTCCTCTCAGAGAAGAG-3′
	R	5′-TGGCTGTGATGGTCAGAGTGACAG-3′
*CREBH*	F	5′-AGTGTTCTCCAGAACTTTGC-3′
	R	5′-TGCACGTCCTGAGCCAGT-3′
*AIbZIP*	F	5′-GATGGGCTGGAGAGCAG-3′
	R	5′-GCAGGATGATGAGAGCCAG-3′
*LUMAN*	F	5′-AAGAGGGGACCCAGATGACT-3′
	R	5′-AGGAGGAGGCAGAAGGAGAC-3′
*PRNP*	F	5′-TACTGATTCGCAGTCATTATGGCGAACCTTGGCTGCTGG-3′
	R	5′-GTACTGAGGATCCTCCTCATCCCACTATCAGGAAGA-3′
*HSPA5*	F	5′-TCAAGTTCTTGCCGTTCAAGG-3′
	R	5′-AAATAAGCCTCAGCGGTTTCTT-3′
*HPRT1*	F	5′-CCTGGCGTGGTGATTAGTGAT-3′
	R	5′-AGACGTTCAGTCCTGTCCATAA-3′
*MAP2*	F	5′-GCAGTTCTCAAAGGCTAGAC-3′
	R	5′-TTGATCGTGGAACTCCATCT-3′
*GFAP*	F	5′-GTGGGCAGGTGGGAGCTTGATTCT-3′
	R	5′-CTGGGGCGGCCTGGTATGACA-3′
*sXBP1*	F	5′-TGCTGAGTCCGCAGCAGGTG-3′
	R	5′-GCTGGCAGGCTCTGGGGAAG-3′
*Luman*	F	5′-TGTGCCCGCGGAGTATGTTG-3′
	R	5′-AGAAGGTTGGAGTCGGAGAA-3′
*Hprt1*	F	5′-GTAATGATCAAGTCAACGGGGGAC-3′
	R	5′-CCAGCAAGCTTGCAACCTTAACCA-3′
*Quantitative PCR*		
*PRNP*	F	5′-AGAGGCCCAGGTCACTCC-3′
	R	5′-GAGCTTCTCCTCTCCTCACG-3′
*LUMAN*	F	OriGene-qStar Primers-H.sapiens CREB3 (Luman/LZIP) #HP209330
	R	
*HPRT1*	F	5′-CCTGGCGTGGTGATTAGTGAT-3′
	R	5′-AGACGTTCAGTCCTGTCCATAA-3′
*Chromatin immunoprecipitation*		
*PRNP*	F	5′-CTGAGCCTTTCATTTTCTCG-3′
	R	5′-GAGATTCGCTTGAACACTTG-3′
*HERPUD1*	F	5′-CAGACGCGGCGGGTTGCA-3′
	R	5′-TCTATAAAAGGCGCCCGAAGC-3′
*ACTB*	F	5′-CTGGAACGGTGAAGGTGACA-3′
	R	5′-AAGGGACTTCCTGTAACAATGCA-3′
