# Supplementary material for: Luman contributes to brefeldin A-induced prion protein gene expression by interacting with the ERSE26 element
Source: Sci Rep. 2017 Feb 13;7:42285. doi: 10.1038/srep42285 (PMC5304227; doi:10.1038/srep42285)
Supplement: Supplementary Information [file srep42285-s1.pdf]

Supplementary Information

Luman contributes to brefeldin A-induced prion protein gene expression by interacting with the ERSE26 element

Marc-André Déry[1,2] and Andréa C. LeBlanc[1,2]

[1]Lady Davis Institute for Medical Research, Sir Mortimer B. Davis Jewish General Hospital,

[2]Department of Neurology and Neurosurgery, McGill University, Montreal, Quebec, Canada.

To whom correspondence should be addressed: Andréa C. LeBlanc (andrea.leblanc@mcgill.ca) The Bloomfield Center for Research in Aging, Lady Davis Institute for Medical Research, The Sir Mortimer B. Davis Jewish General Hospital, 3755 ch. de la Côte Sainte Catherine, Montréal, Québec, Canada, H3T 1E2. Telephone: 514-340-8222 ext:4976. Fax: 514-340-8295.

Figure 1a

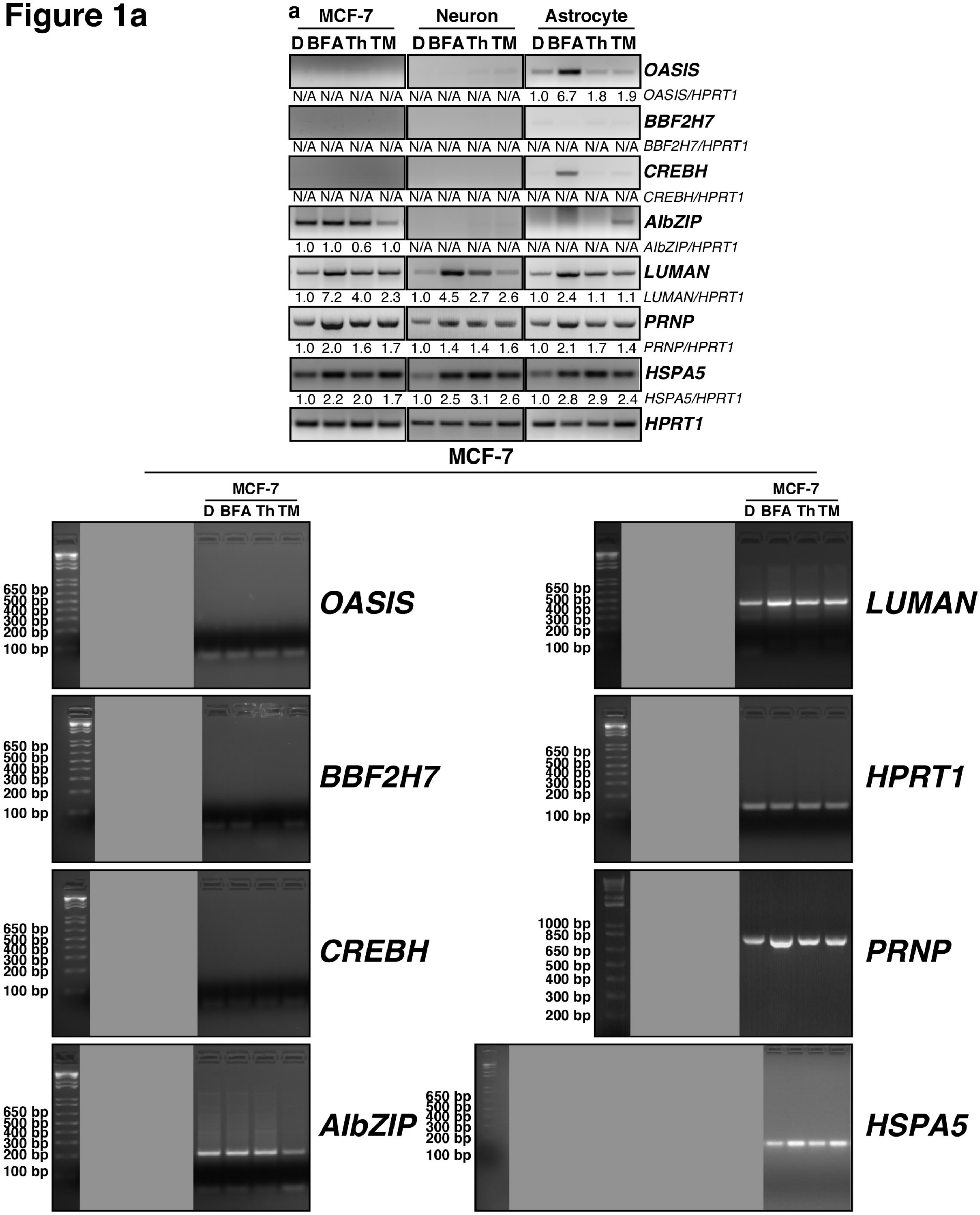

a
MCF-7
Neuron
Astrocyte
D BFA Th TM
OASIS
N/A N/A N/A N/A N/A N/A N/A N/A 1.0 6.7 1.8 1.9 OASIS/HPRT1
BBF2H7
N/A N/A N/A N/A N/A N/A N/A N/A N/A N/A N/A N/A BBF2H7/HPRT1
CREBH
N/A N/A N/A N/A N/A N/A N/A N/A N/A N/A N/A N/A CREBH/HPRT1
AIbZIP
1.0 1.0 0.6 1.0 N/A N/A N/A N/A N/A N/A N/A N/A AIbZIP/HPRT1
LUMAN
1.0 7.2 4.0 2.3 1.0 4.5 2.7 2.6 1.0 2.4 1.1 1.1 LUMAN/HPRT1
PRNP
1.0 2.0 1.6 1.7 1.0 1.4 1.4 1.6 1.0 2.1 1.7 1.4 PRNP/HPRT1
HSPA5
1.0 2.2 2.0 1.7 1.0 2.5 3.1 2.6 1.0 2.8 2.9 2.4 HSPA5/HPRT1
HPRT1
MCF-7
650 bp
500 bp
400 bp
300 bp
200 bp
100 bp
OASIS
BBF2H7
CREBH
AIbZIP
LUMAN
HPRT1
1000 bp
850 bp
PRNP
HSPA5

Figure 1a

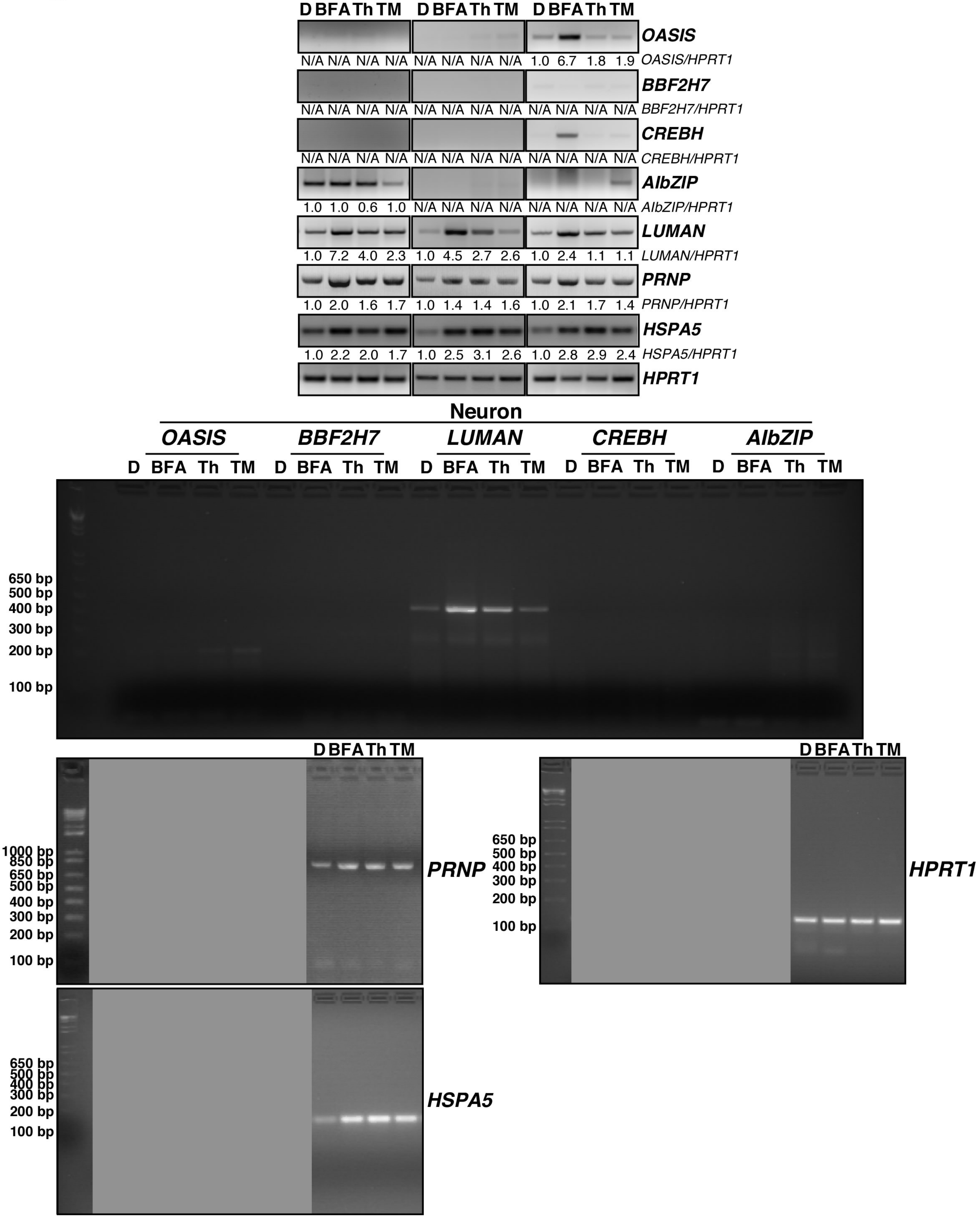
a
MCF-7
Neuron
Astrocyte
D BFA Th TM D BFA Th TM D BFA Th TM
OASIS
N/A N/A N/A N/A N/A N/A N/A N/A 1.0 6.7 1.8 1.9 OASIS/HPRT1
BBF2H7
N/A N/A N/A N/A N/A N/A N/A N/A N/A N/A N/A N/A BBF2H7/HPRT1
CREBH
N/A N/A N/A N/A N/A N/A N/A N/A N/A N/A N/A N/A CREBH/HPRT1
AIbZIP
1.0 1.0 0.6 1.0 N/A N/A N/A N/A N/A N/A N/A N/A AIbZIP/HPRT1
LUMAN
1.0 7.2 4.0 2.3 1.0 4.5 2.7 2.6 1.0 2.4 1.1 1.1 LUMAN/HPRT1
PRNP
1.0 2.0 1.6 1.7 1.0 1.4 1.4 1.6 1.0 2.1 1.7 1.4 PRNP/HPRT1
HSPA5
1.0 2.2 2.0 1.7 1.0 2.5 3.1 2.6 1.0 2.8 2.9 2.4 HSPA5/HPRT1
HPRT1
Neuron
OASIS
BBF2H7
LUMAN
CREBH
AIbZIP
D BFA Th TM D BFA Th TM D BFA Th TM D BFA Th TM D BFA Th TM
650 bp
500 bp
400 bp
300 bp
200 bp
100 bp
D BFA Th TM
1000 bp
850 bp
650 bp
500 bp
400 bp
300 bp
200 bp
100 bp
PRNP
D BFA Th TM
650 bp
500 bp
400 bp
300 bp
200 bp
100 bp
HPRT1
650 bp
500 bp
400 bp
300 bp
200 bp
100 bp
HSPA5

Figure 1a

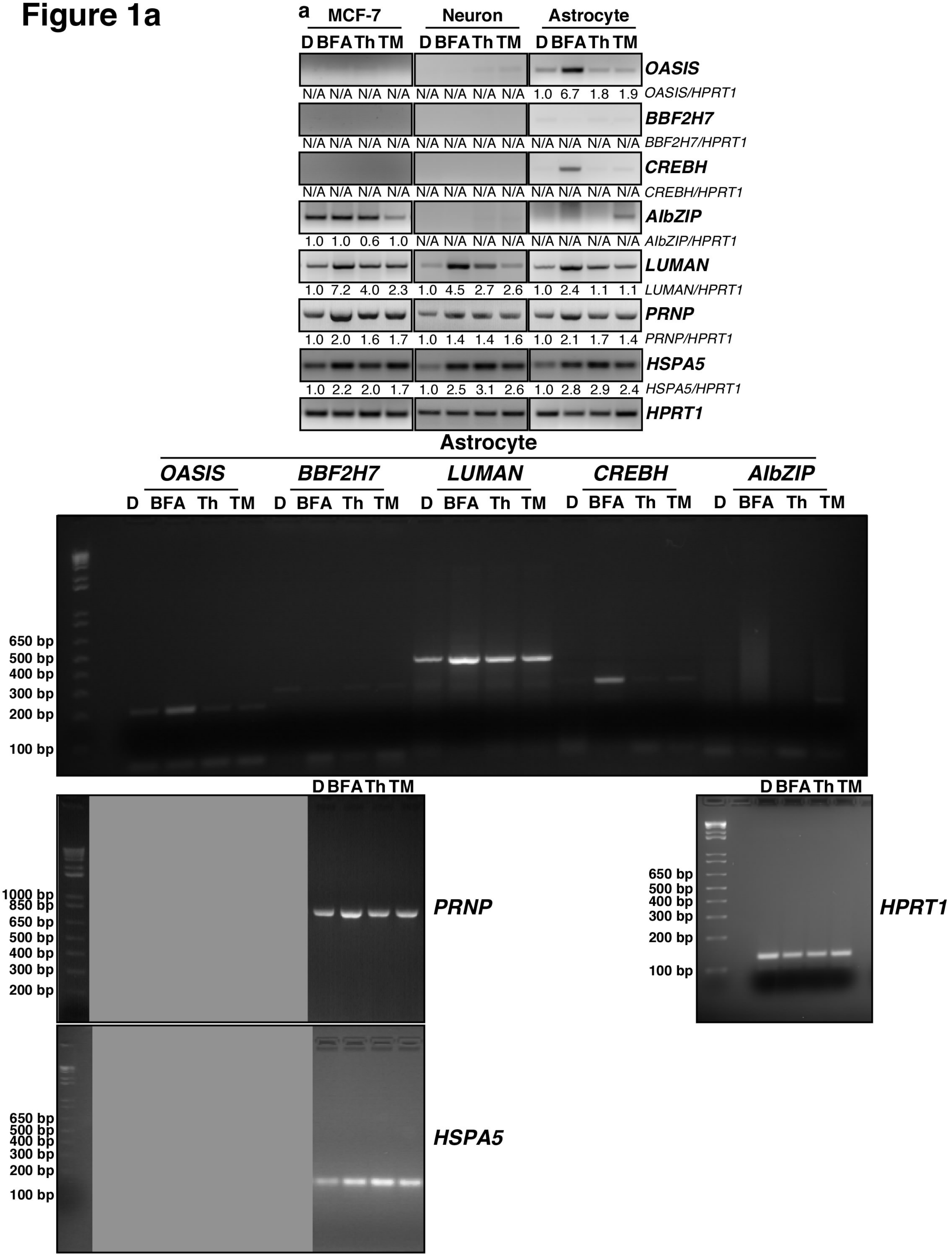
a
MCF-7
Neuron
Astrocyte
D BFA Th TM D BFA Th TM D BFA Th TM
OASIS
N/A N/A N/A N/A N/A N/A N/A N/A 1.0 6.7 1.8 1.9 OASIS/HPRT1
BBF2H7
N/A N/A N/A N/A N/A N/A N/A N/A N/A N/A N/A N/A BBF2H7/HPRT1
CREBH
N/A N/A N/A N/A N/A N/A N/A N/A N/A N/A N/A N/A CREBH/HPRT1
AIbZIP
1.0 1.0 0.6 1.0 N/A N/A N/A N/A N/A N/A N/A N/A AIbZIP/HPRT1
LUMAN
1.0 7.2 4.0 2.3 1.0 4.5 2.7 2.6 1.0 2.4 1.1 1.1 LUMAN/HPRT1
PRNP
1.0 2.0 1.6 1.7 1.0 1.4 1.4 1.6 1.0 2.1 1.7 1.4 PRNP/HPRT1
HSPA5
1.0 2.2 2.0 1.7 1.0 2.5 3.1 2.6 1.0 2.8 2.9 2.4 HSPA5/HPRT1
HPRT1
Astrocyte
OASIS
BBF2H7
LUMAN
CREBH
AIbZIP
D BFA Th TM D BFA Th TM D BFA Th TM D BFA Th TM D BFA Th TM
650 bp
500 bp
400 bp
300 bp
200 bp
100 bp
D BFA Th TM
1000 bp
850 bp
650 bp
500 bp
400 bp
300 bp
200 bp
PRNP
650 bp
500 bp
400 bp
300 bp
200 bp
100 bp
HSPA5
D BFA Th TM
650 bp
500 bp
400 bp
300 bp
200 bp
100 bp
HPRT1

Figure 1d

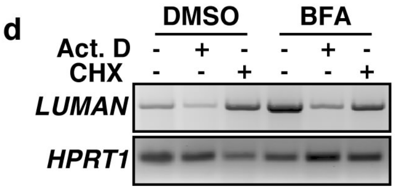
d
DMSO
BFA
Act. D - + - - + -
CHX - - + - - +
LUMAN
HPRT1

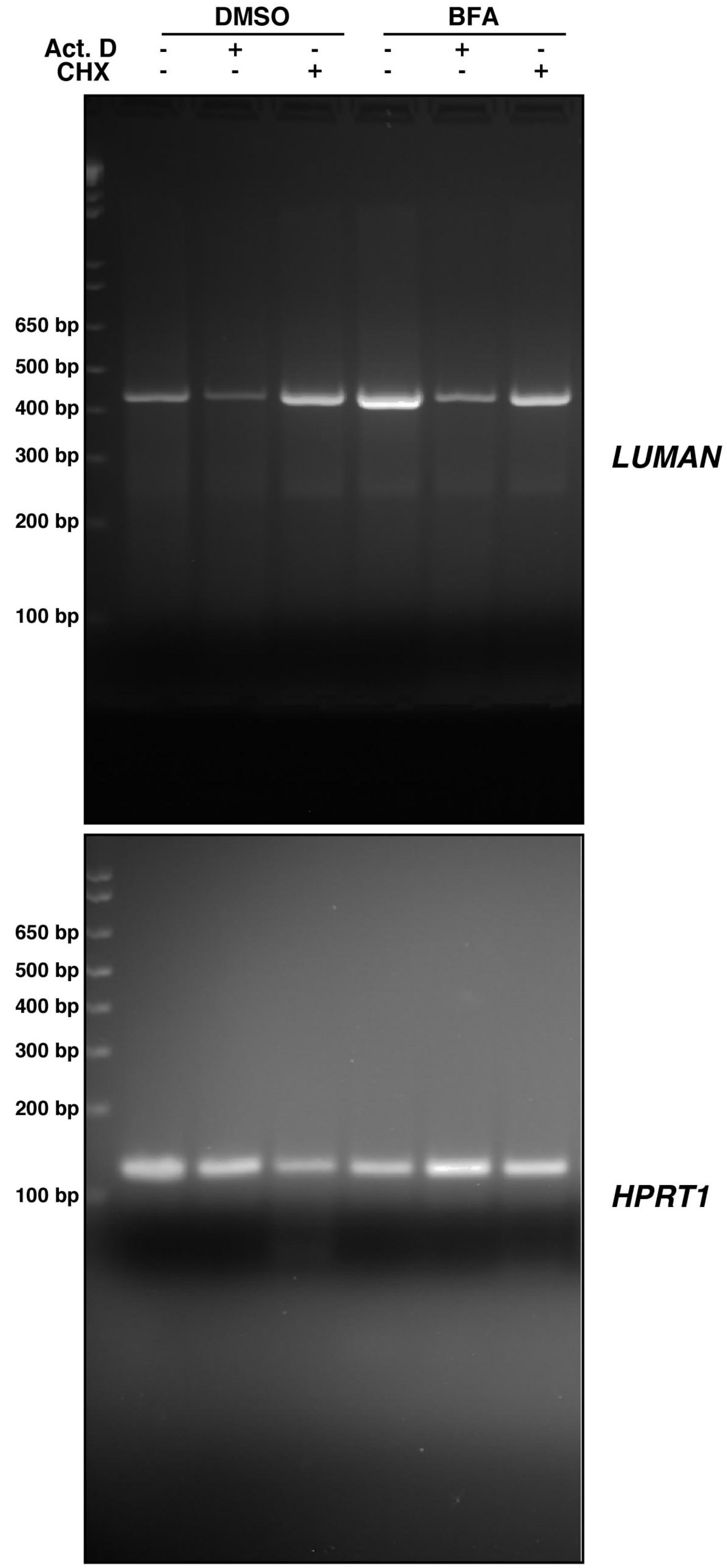
DMSO
BFA
Act. D - + - - + -
CHX - - + - - +
650 bp
500 bp
400 bp
300 bp
200 bp
100 bp
LUMAN
650 bp
500 bp
400 bp
300 bp
200 bp
100 bp
HPRT1

Figure 1e

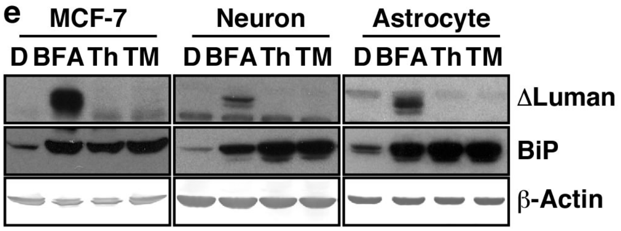
e
MCF-7
Neuron
Astrocyte
D BFA Th TM
D BFA Th TM
D BFA Th TM
ΔLuman
BiP
β-Actin

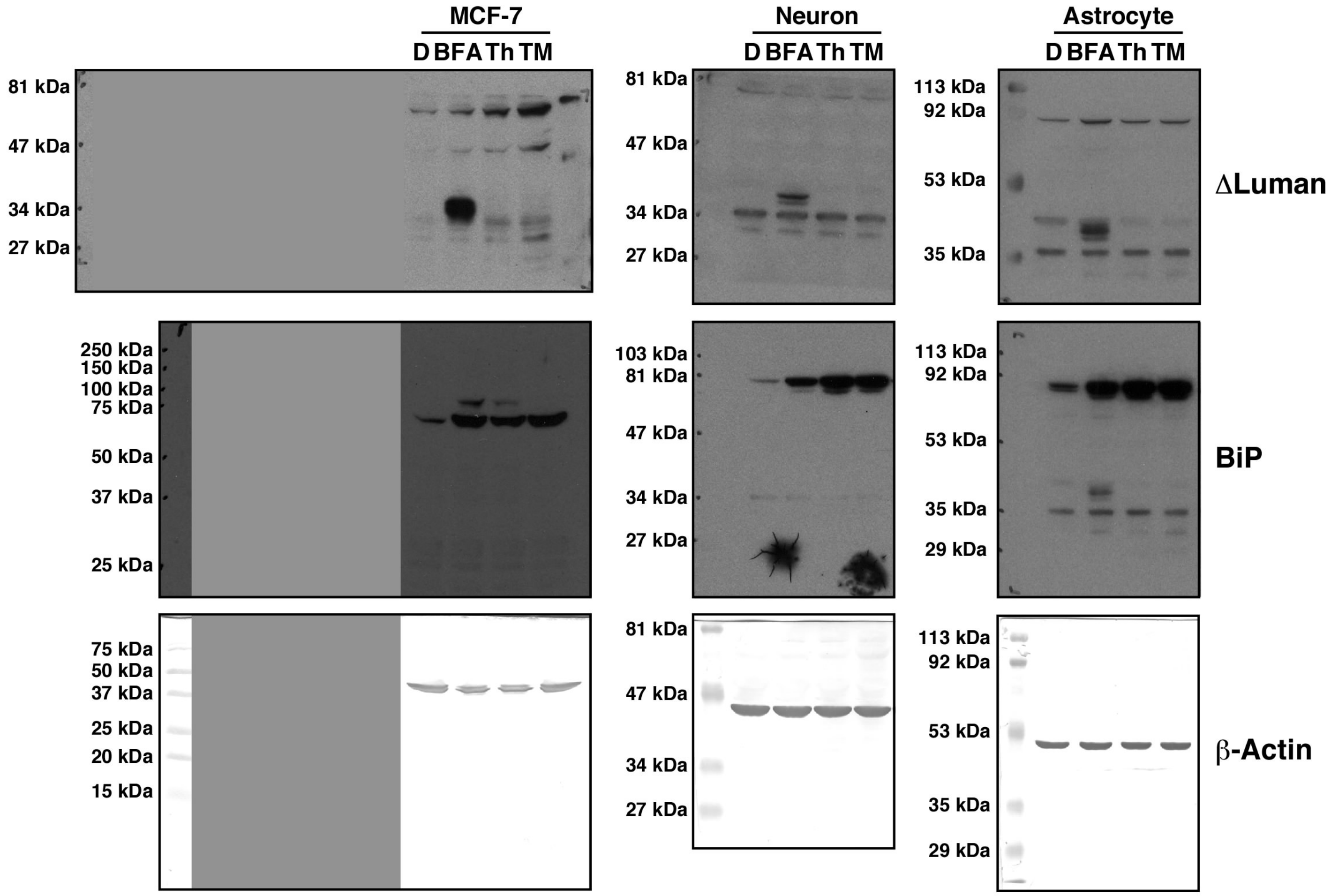
MCF-7
D BFA Th TM
Neuron
D BFA Th TM
Astrocyte
D BFA Th TM
81 kDa
47 kDa
34 kDa
27 kDa
81 kDa
47 kDa
34 kDa
27 kDa
113 kDa
92 kDa
53 kDa
35 kDa
ΔLuman
250 kDa
150 kDa
100 kDa
75 kDa
50 kDa
37 kDa
25 kDa
103 kDa
81 kDa
47 kDa
34 kDa
27 kDa
113 kDa
92 kDa
53 kDa
35 kDa
29 kDa
BiP
75 kDa
50 kDa
37 kDa
25 kDa
20 kDa
15 kDa
81 kDa
47 kDa
34 kDa
27 kDa
113 kDa
92 kDa
53 kDa
35 kDa
29 kDa
β-Actin

Figure 1f

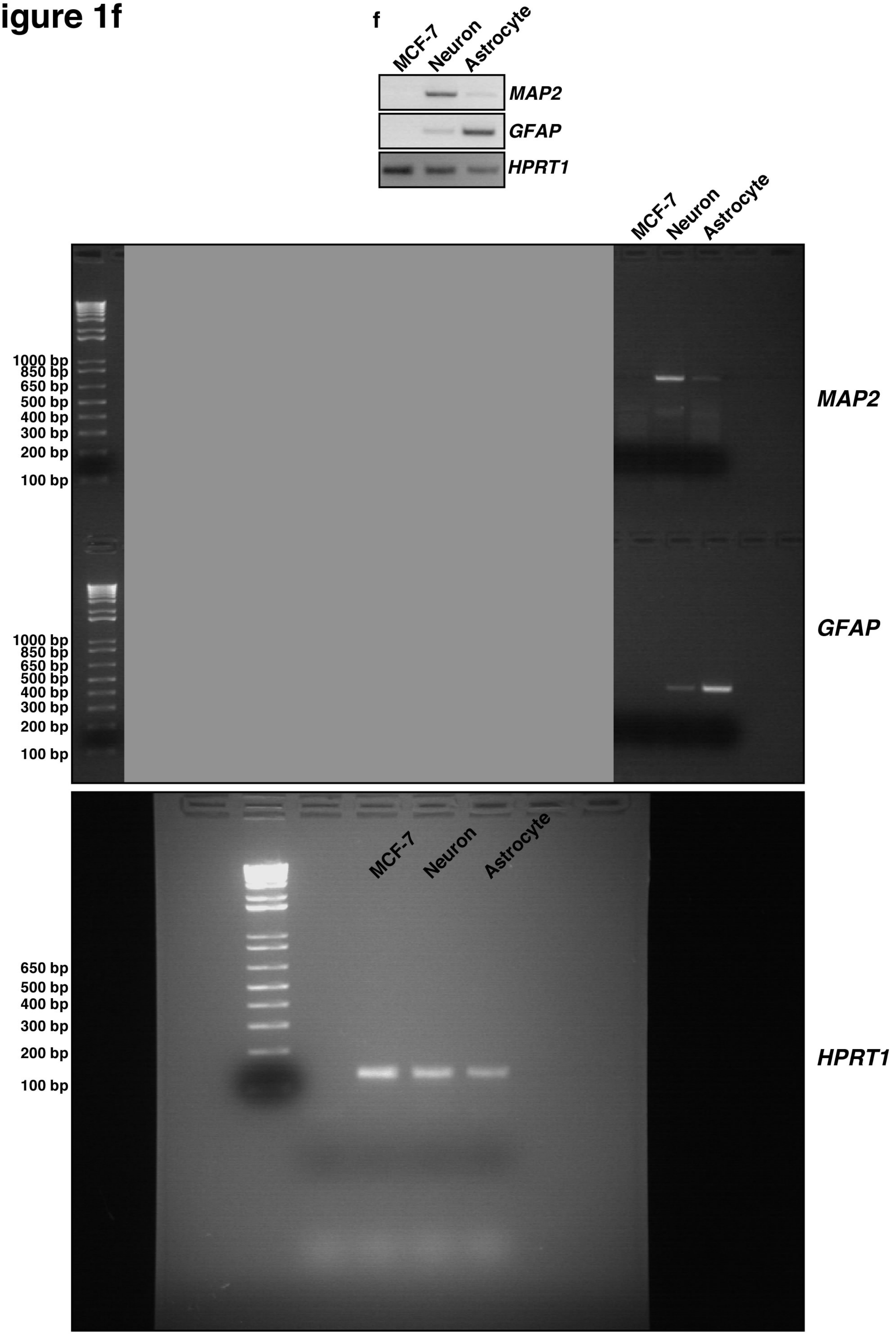

f
MCF-7
Neuron
Astrocyte
MAP2
GFAP
HPRT1
MCF-7
Neuron
Astrocyte
1000 bp
850 bp
650 bp
500 bp
400 bp
300 bp
200 bp
100 bp
MAP2
1000 bp
850 bp
650 bp
500 bp
400 bp
300 bp
200 bp
100 bp
GFAP
MCF-7
Neuron
Astrocyte
650 bp
500 bp
400 bp
300 bp
200 bp
100 bp
HPRT1

Figure 1g

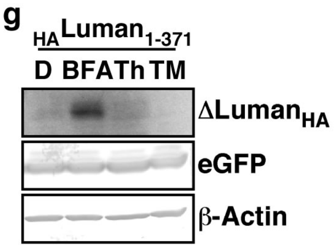
g
HALuman1-371
D BFA Th TM
ΔLumanHA
eGFP
β-Actin

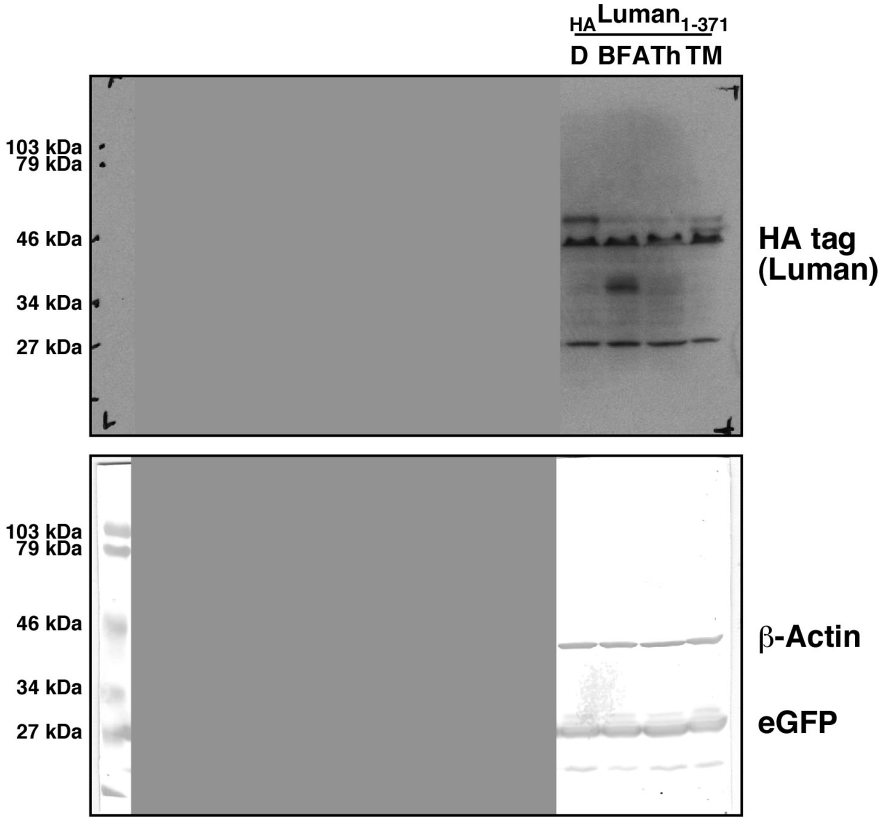
HALuman1-371
D BFA Th TM
103 kDa
79 kDa
46 kDa
34 kDa
27 kDa
HA tag
(Luman)
103 kDa
79 kDa
46 kDa
34 kDa
27 kDa
β-Actin
eGFP

Figure 1h

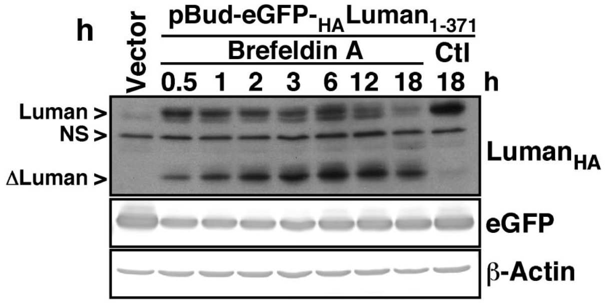

h
Vector
pBud-eGFP-HALuman1-371
Brefeldin A
Ctl
0.5 1 2 3 6 12 18 18 h
Luman >
NS >
ΔLuman >
LumanHA
eGFP
β-Actin

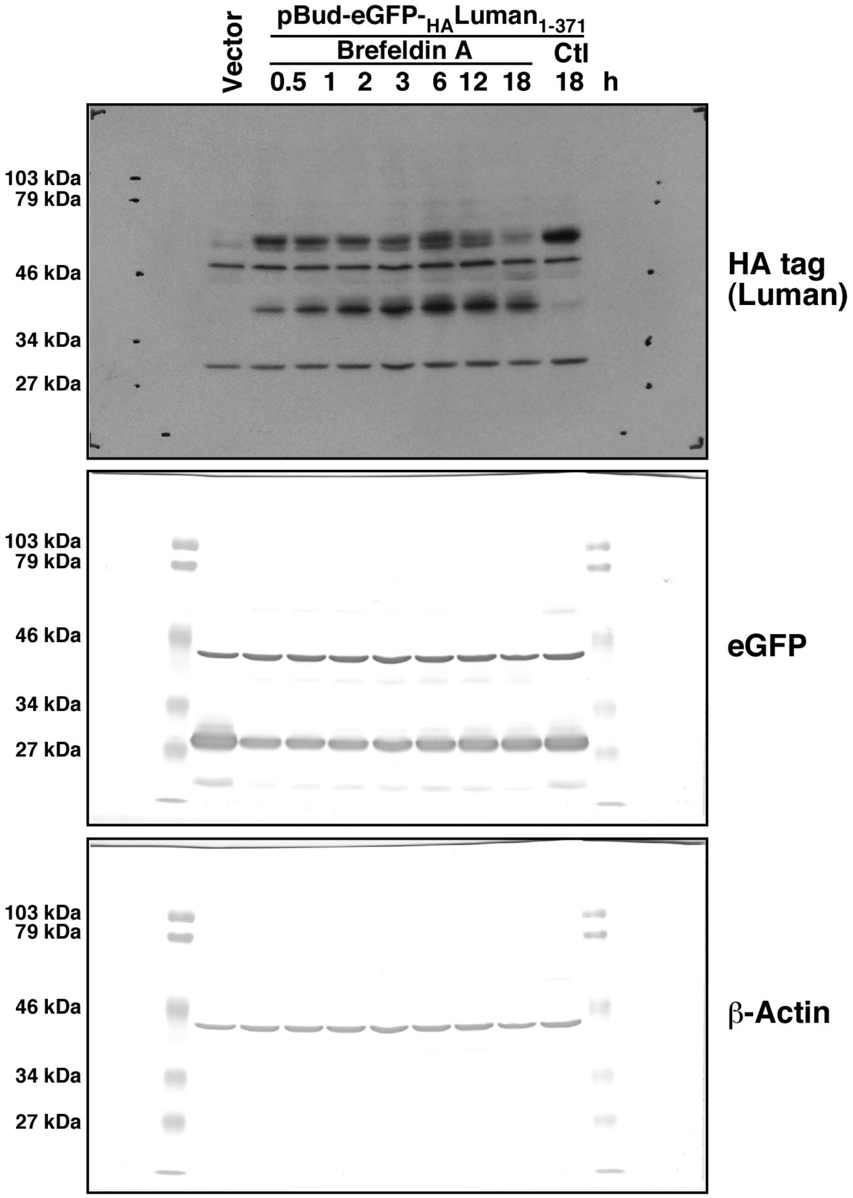

Vector
pBud-eGFP-HALuman1-371
Brefeldin A
Ctl
0.5 1 2 3 6 12 18 18 h
103 kDa
79 kDa
46 kDa
34 kDa
27 kDa
HA tag
(Luman)
eGFP
β-Actin

Figure 2a

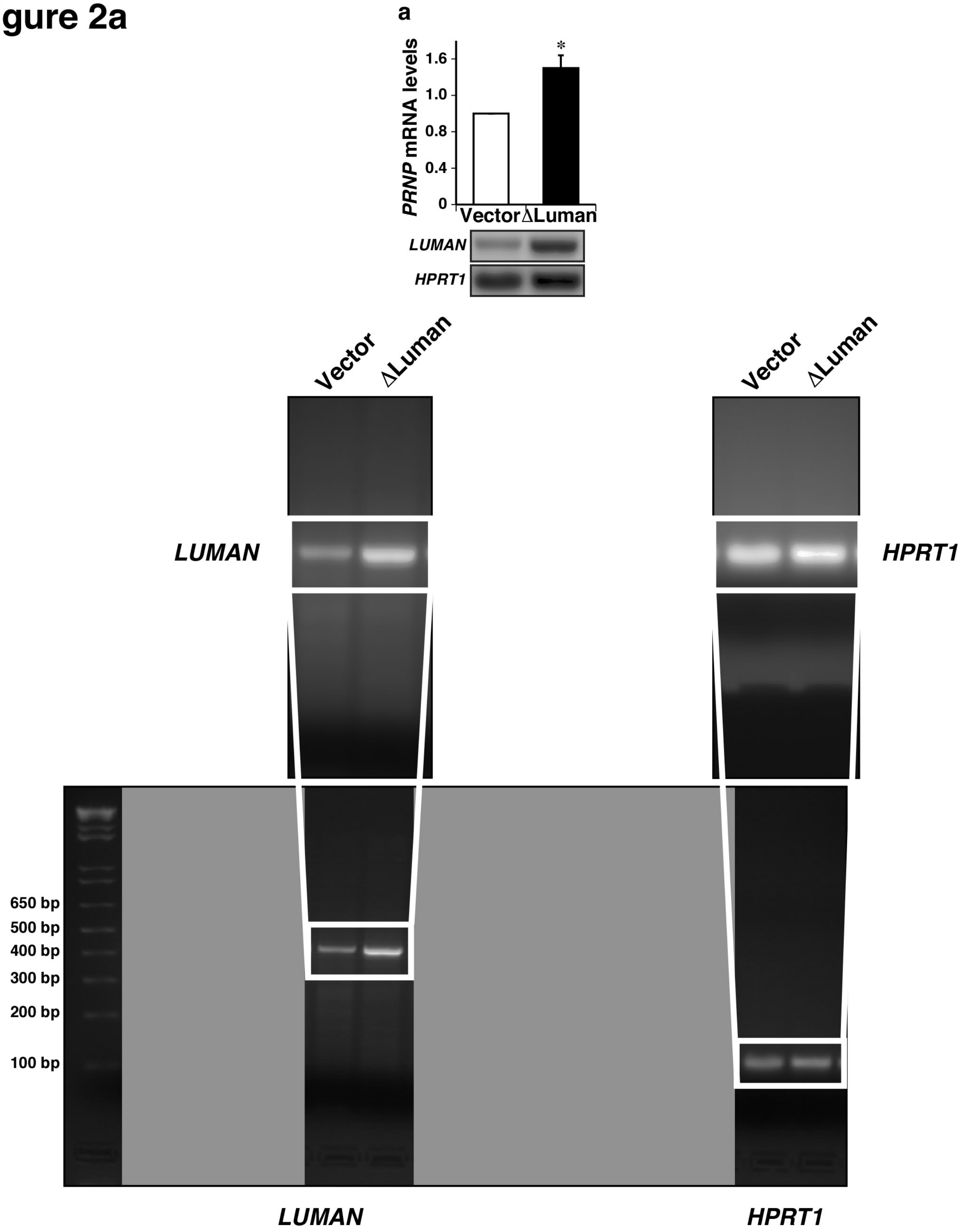

a
PRNP mRNA levels
1.6
1.0
0.8
0.4
0
*
Vector
ΔLuman
LUMAN
HPRT1
Vector
ΔLuman
Vector
ΔLuman
LUMAN
HPRT1
650 bp
500 bp
400 bp
300 bp
200 bp
100 bp
LUMAN
HPRT1

Figure 2b

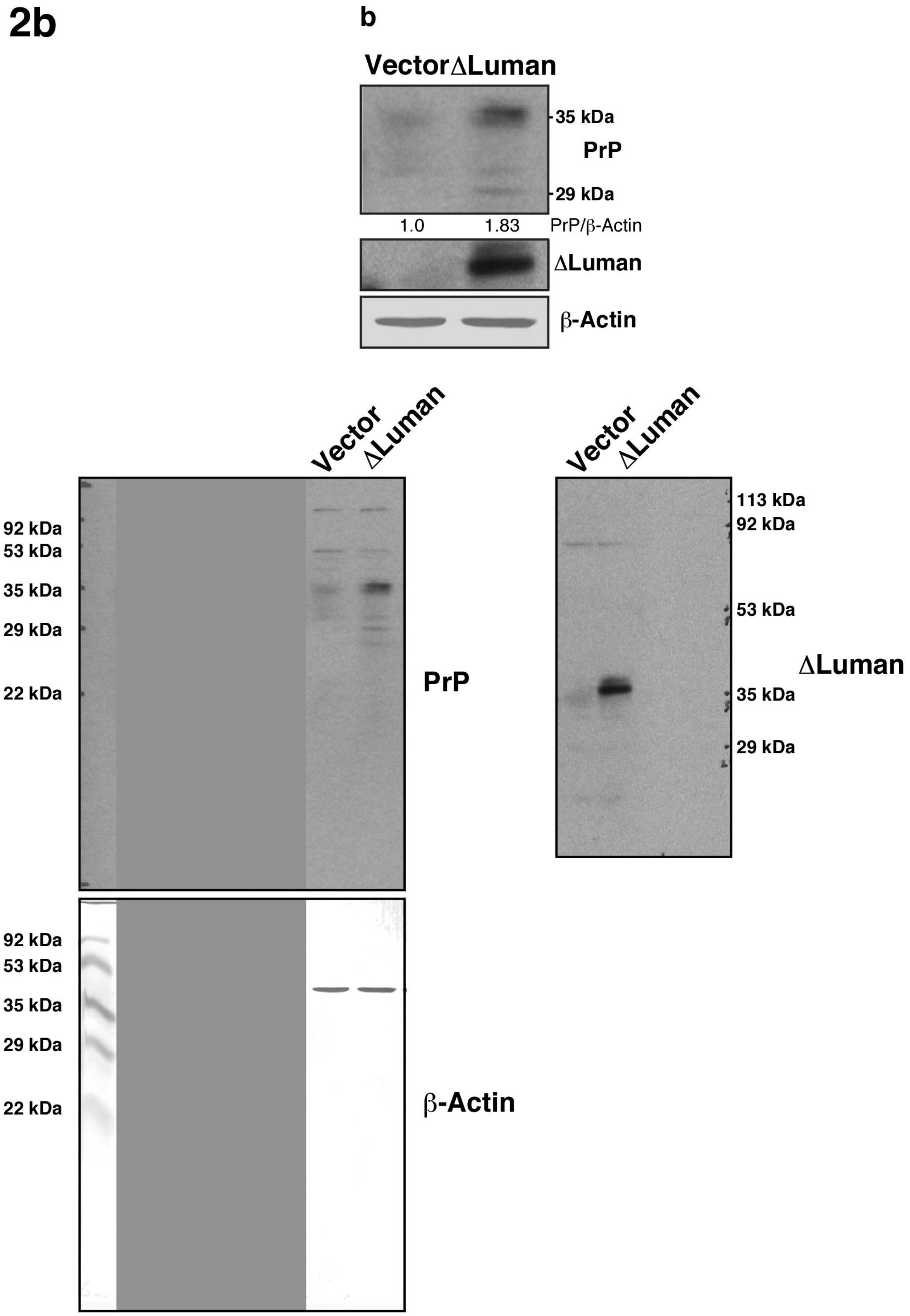
b
Vector ΔLuman
35 kDa
PrP
29 kDa
1.0
1.83
PrP/β-Actin
ΔLuman
β-Actin
Vector
ΔLuman
92 kDa
53 kDa
35 kDa
29 kDa
22 kDa
PrP
92 kDa
53 kDa
35 kDa
29 kDa
22 kDa
β-Actin
Vector
ΔLuman
113 kDa
92 kDa
53 kDa
ΔLuman
35 kDa
29 kDa

Figure 2c

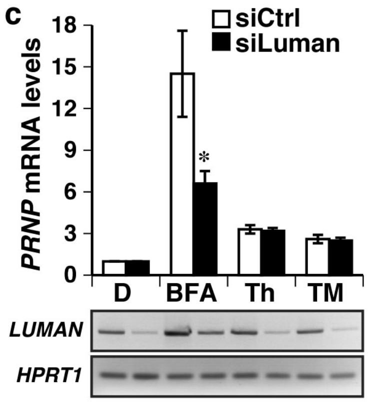
c
siCtrl
siLuman
PRNP mRNA levels
18
15
12
9
6
3
0
*
D
BFA
Th
TM
LUMAN
HPRT1

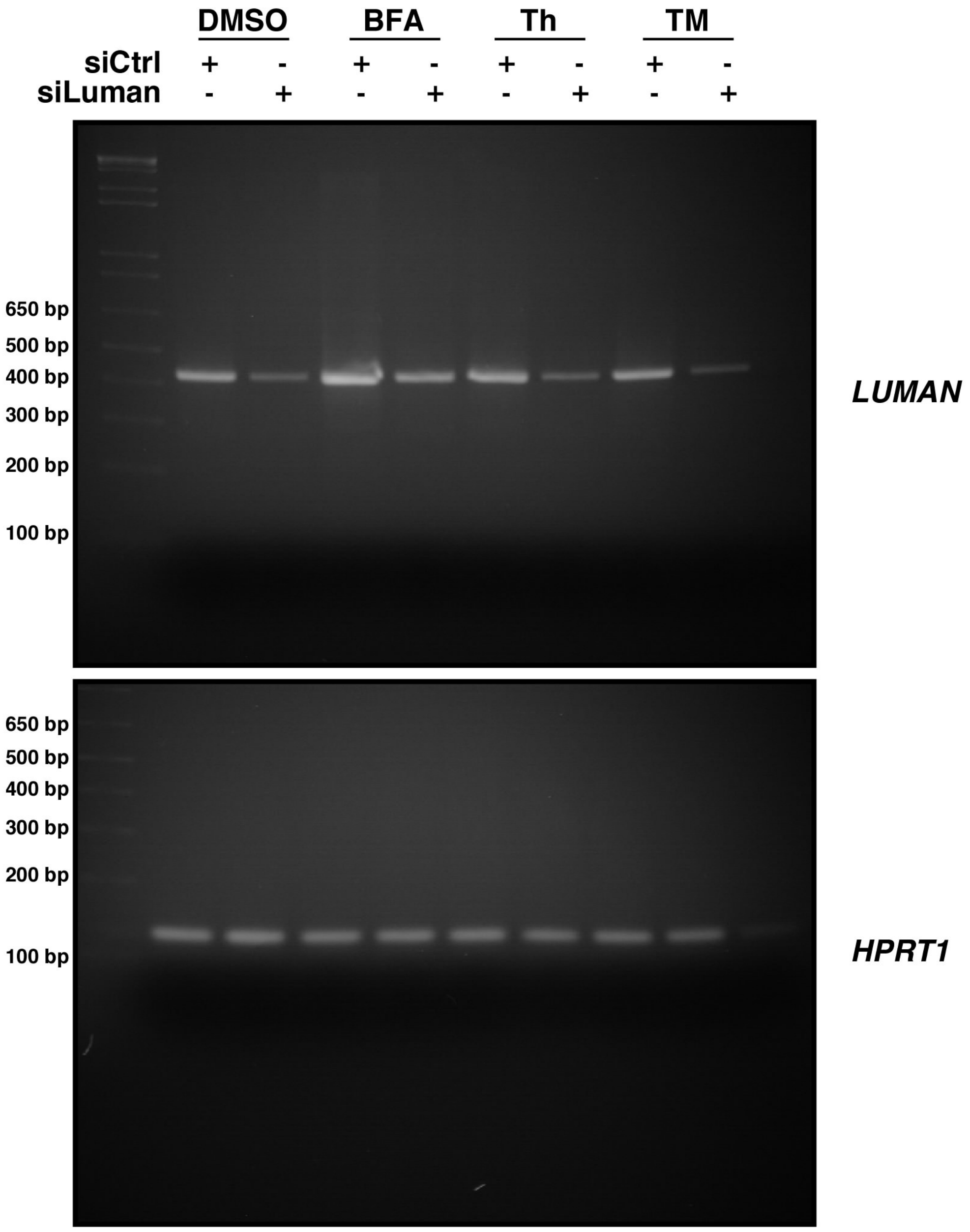
DMSO
BFA
Th
TM
siCtrl + - + - + - + -
siLuman - + - + - + - +
650 bp
500 bp
400 bp
300 bp
200 bp
100 bp
LUMAN
650 bp
500 bp
400 bp
300 bp
200 bp
100 bp
HPRT1

Figure 2d

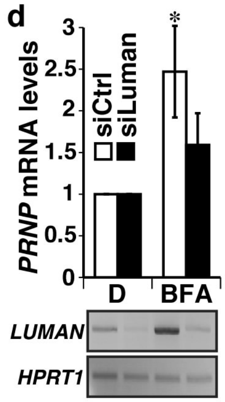
d
PRNP mRNA levels
siCtrl
siLuman
3
2.5
2
1.5
1
0.5
0
*
D
BFA
LUMAN
HPRT1

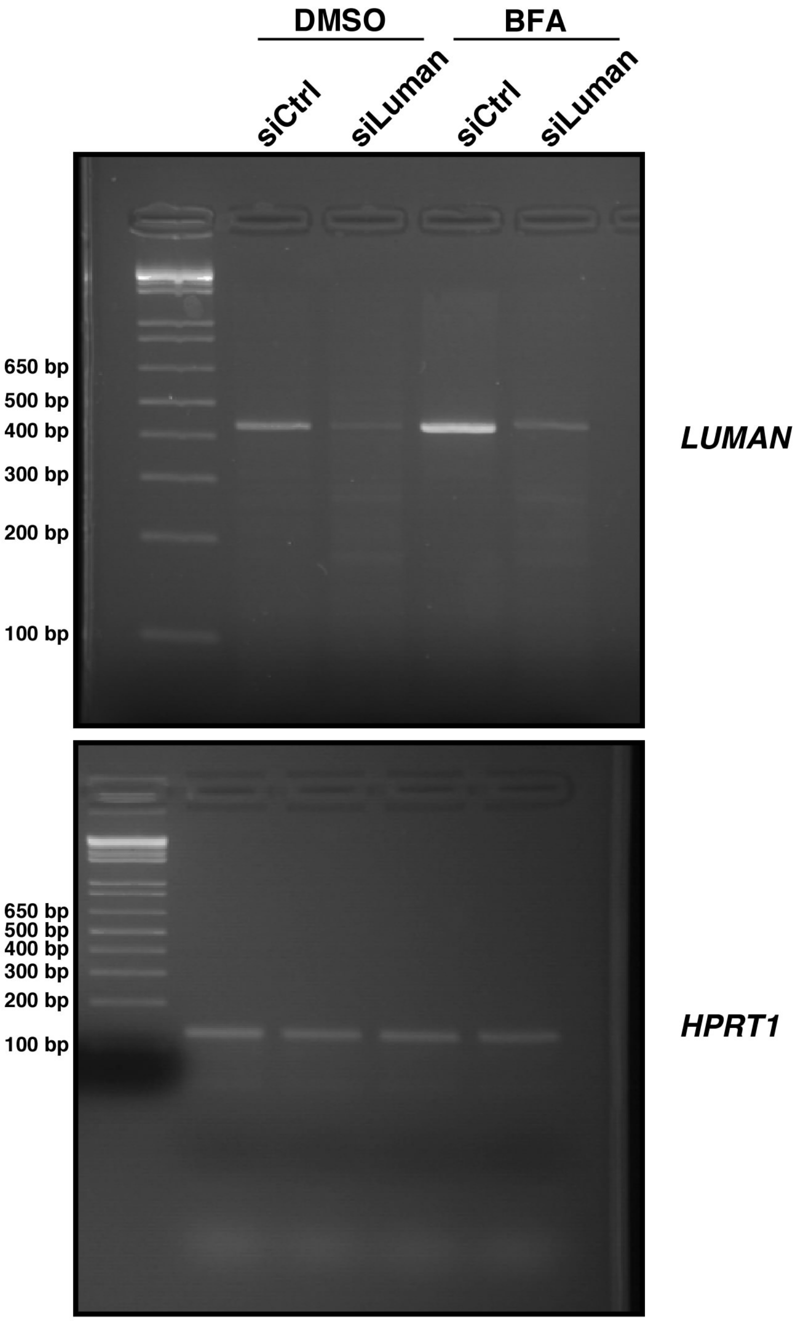
DMSO
BFA
siCtrl
siLuman
siCtrl
siLuman
650 bp
500 bp
400 bp
300 bp
200 bp
100 bp
LUMAN
650 bp
500 bp
400 bp
300 bp
200 bp
100 bp
HPRT1

Figure 2e

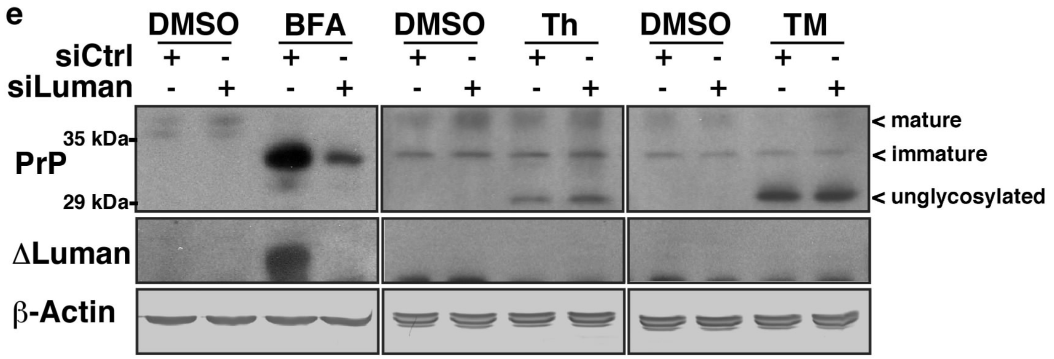

e
DMSO
BFA
DMSO
Th
DMSO
TM
siCtrl
siLuman
35 kDa
29 kDa
PrP
< mature
< immature
< unglycosylated
ΔLuman
β-Actin

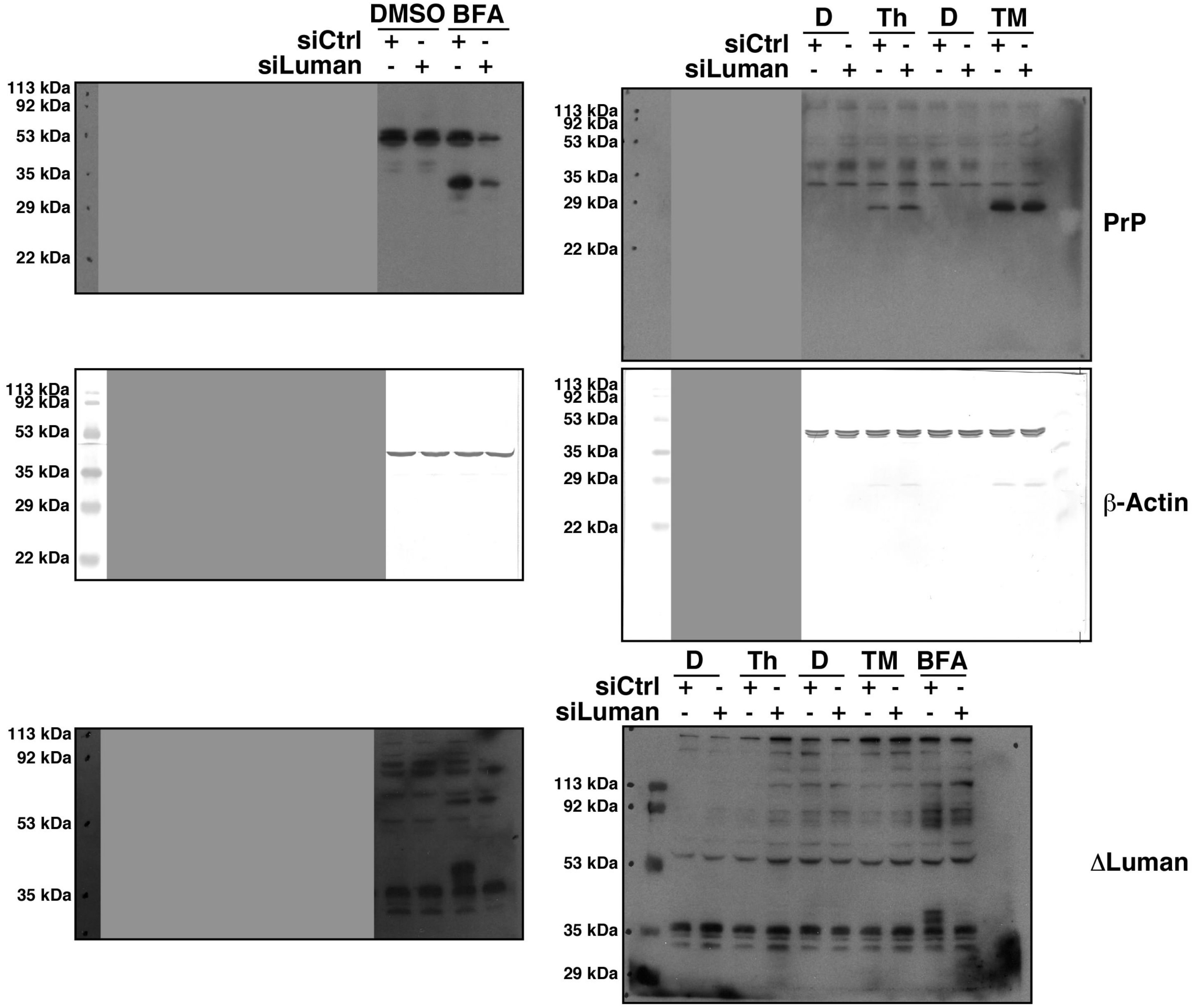

DMSO
BFA
siCtrl
siLuman
D
Th
D
TM
113 kDa
92 kDa
53 kDa
35 kDa
29 kDa
22 kDa
PrP
β-Actin
BFA
ΔLuman

Figure 3a

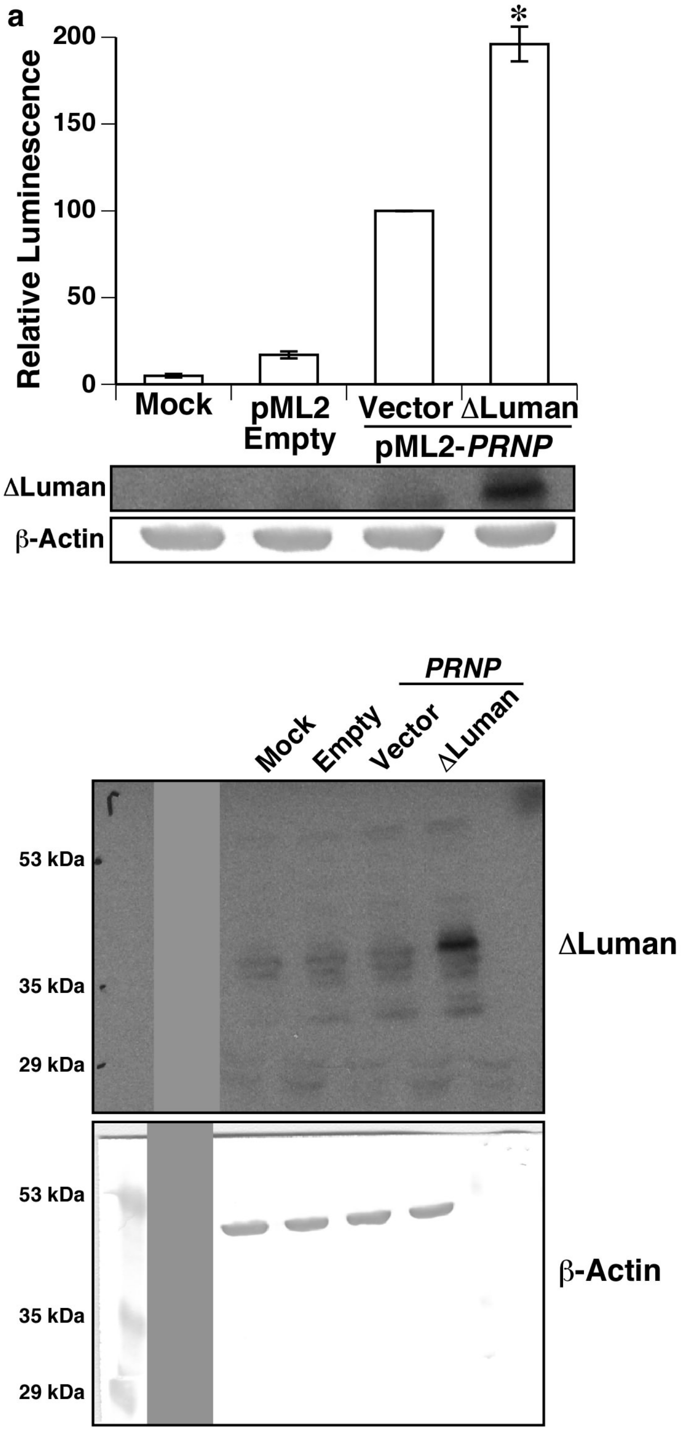
a
Relative Luminescence
200
150
100
50
0
*
Mock
pML2
Empty
Vector
ΔLuman
pML2-PRNP
ΔLuman
β-Actin
PRNP
Mock
Empty
Vector
ΔLuman
53 kDa
35 kDa
29 kDa
ΔLuman
53 kDa
35 kDa
29 kDa
β-Actin

Figure 3c

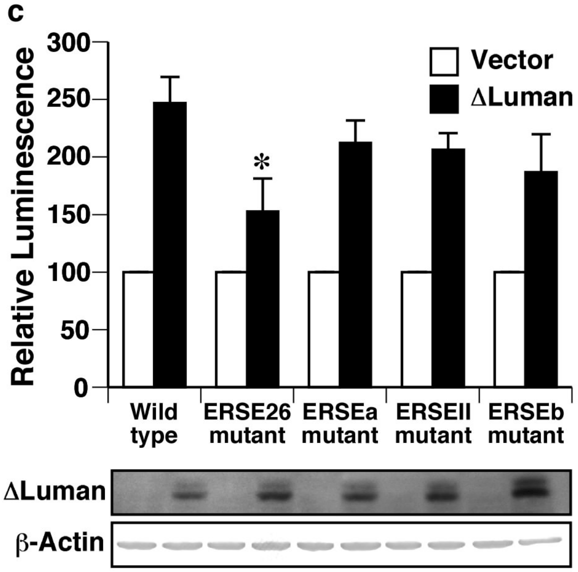
c
Relative Luminescence
300
250
200
150
100
50
0
Vector
ΔLuman
*
Wild type
ERSE26 mutant
ERSEa mutant
ERSEII mutant
ERSEb mutant
ΔLuman
β-Actin

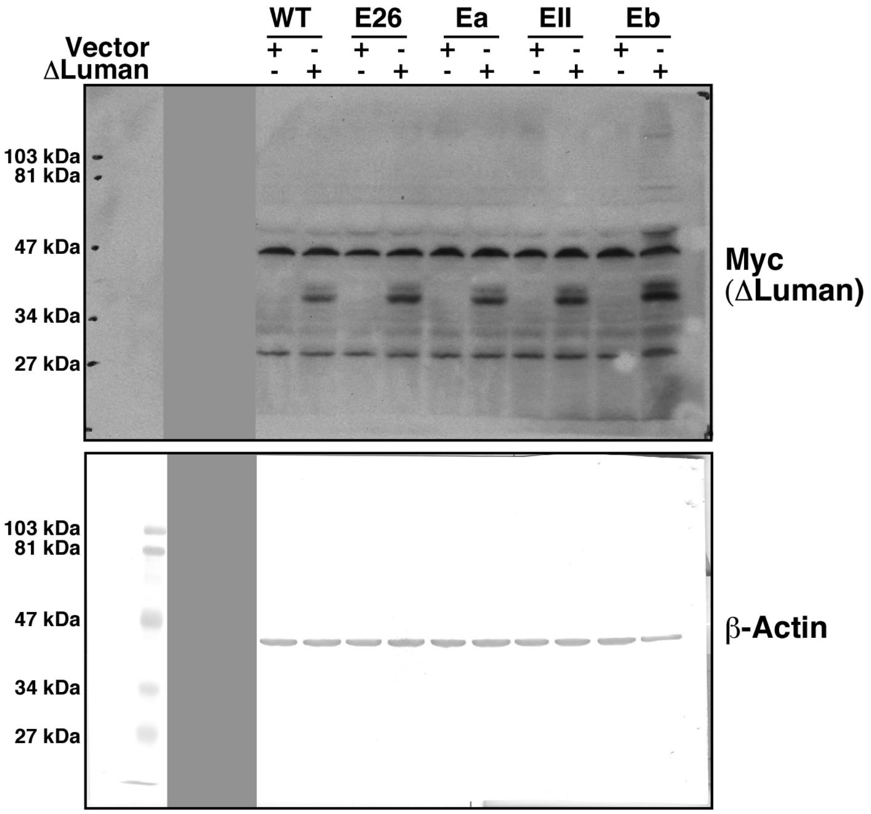
WT
E26
Ea
EII
Eb
Vector
ΔLuman
+ - + - + - + - + -
- + - + - + - + - +
103 kDa
81 kDa
47 kDa
34 kDa
27 kDa
Myc (ΔLuman)
103 kDa
81 kDa
47 kDa
34 kDa
27 kDa
β-Actin

Figure 3d

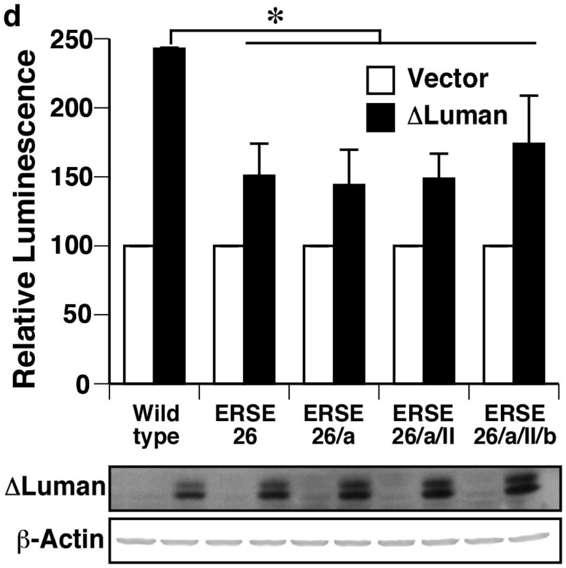
d
*
Relative Luminescence
250
200
150
100
50
0
Vector
ΔLuman
Wild type
ERSE 26
ERSE 26/a
ERSE 26/a/II
ERSE 26/a/II/b
ΔLuman
β-Actin

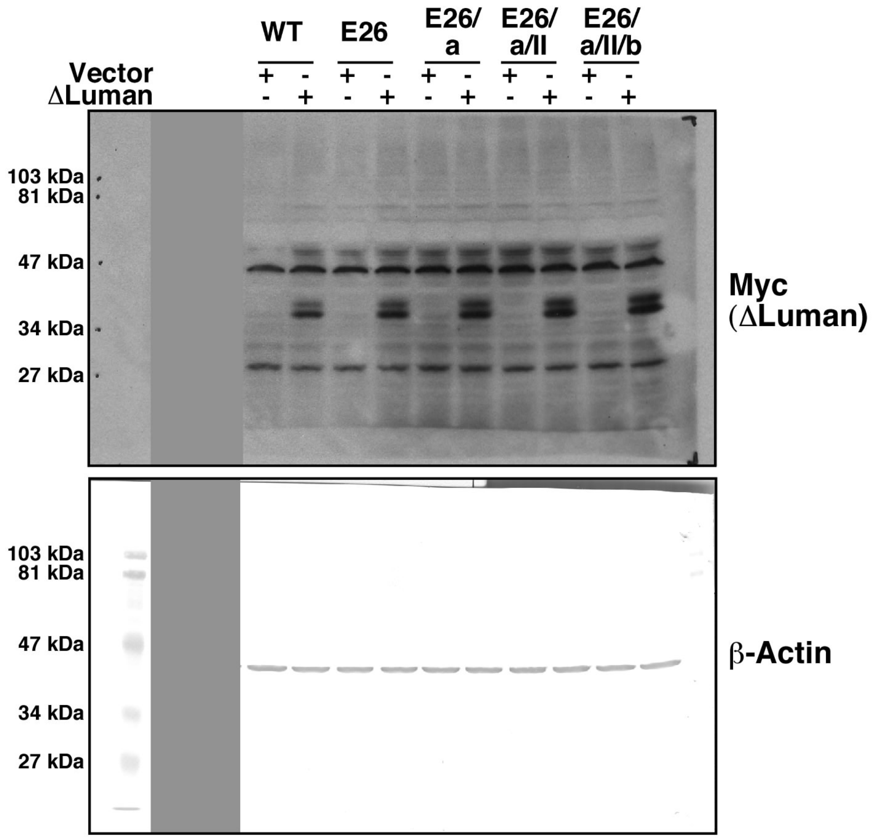
WT
E26
E26/ a
E26/ a/II
E26/ a/II/b
Vector
ΔLuman
+ - + - + - + - + -
- + - + - + - + - +
103 kDa
81 kDa
47 kDa
34 kDa
27 kDa
Myc (ΔLuman)
103 kDa
81 kDa
47 kDa
34 kDa
27 kDa
β-Actin

Figure 3e

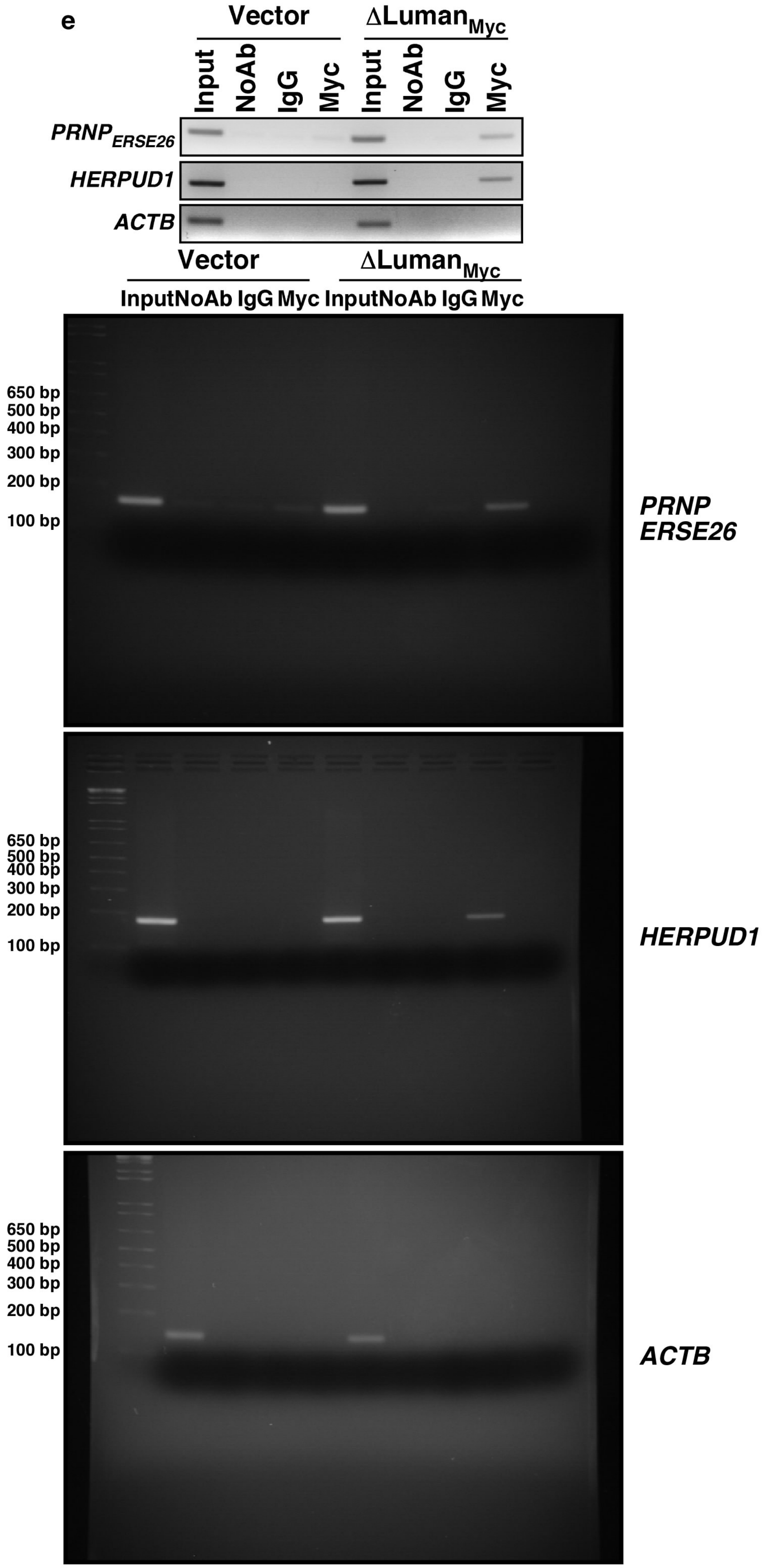

e
Vector
ΔLuman Myc
Input
NoAb
IgG
Myc
Input
NoAb
IgG
Myc
PRNP ERSE26
HERPUD1
ACTB
Vector
ΔLuman Myc
Input NoAb IgG Myc Input NoAb IgG Myc
650 bp
500 bp
400 bp
300 bp
200 bp
100 bp
PRNP
ERSE26
650 bp
500 bp
400 bp
300 bp
200 bp
100 bp
HERPUD1
650 bp
500 bp
400 bp
300 bp
200 bp
100 bp
ACTB

Figure 3f

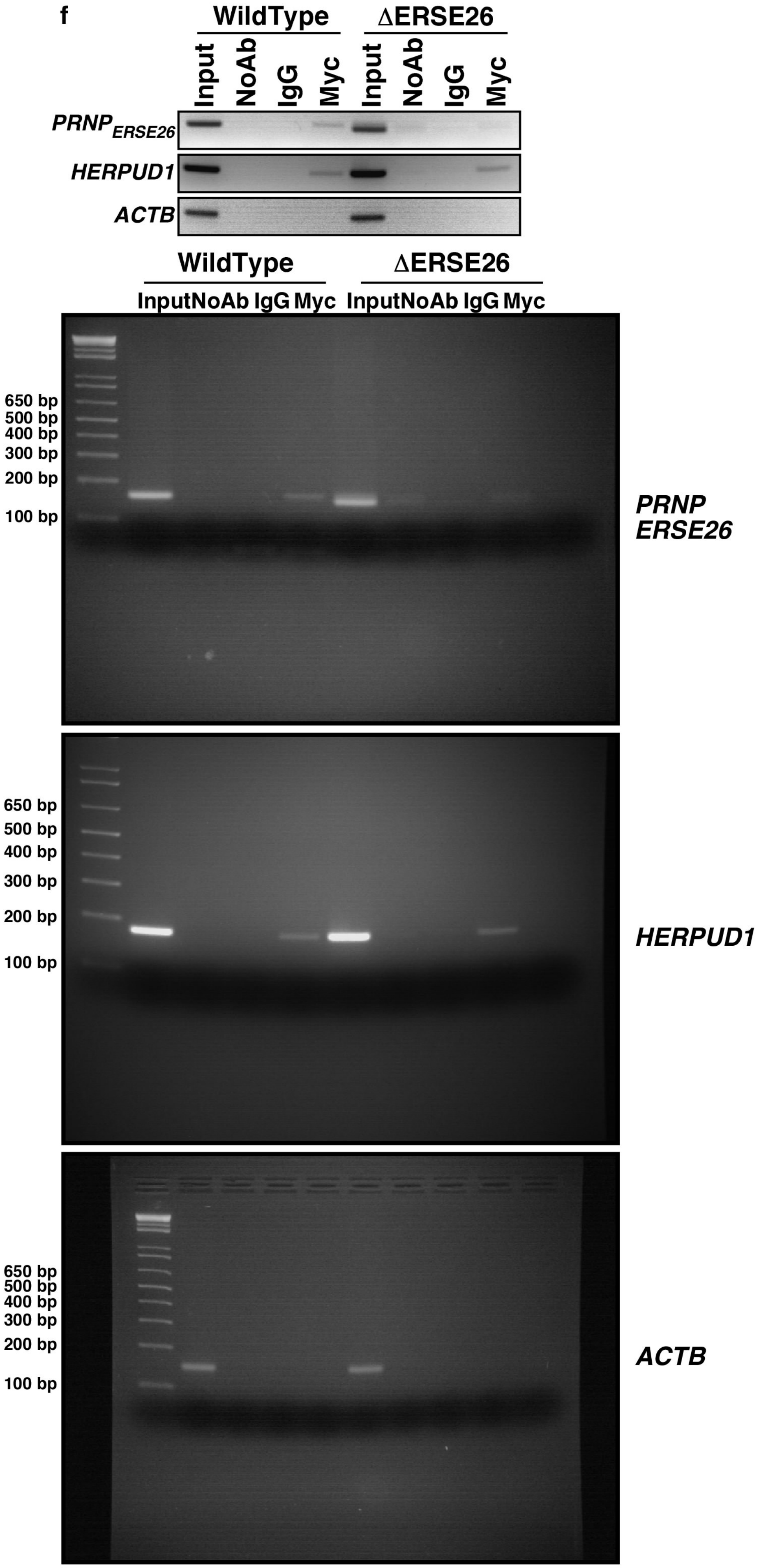
f
WildType
ΔERSE26
Input
NoAb
IgG
Myc
Input
NoAb
IgG
Myc
PRNP ERSE26
HERPUD1
ACTB
WildType
ΔERSE26
Input NoAb IgG Myc Input NoAb IgG Myc
650 bp
500 bp
400 bp
300 bp
200 bp
100 bp
PRNP
ERSE26
650 bp
500 bp
400 bp
300 bp
200 bp
100 bp
HERPUD1
650 bp
500 bp
400 bp
300 bp
200 bp
100 bp
ACTB

Figure 4a

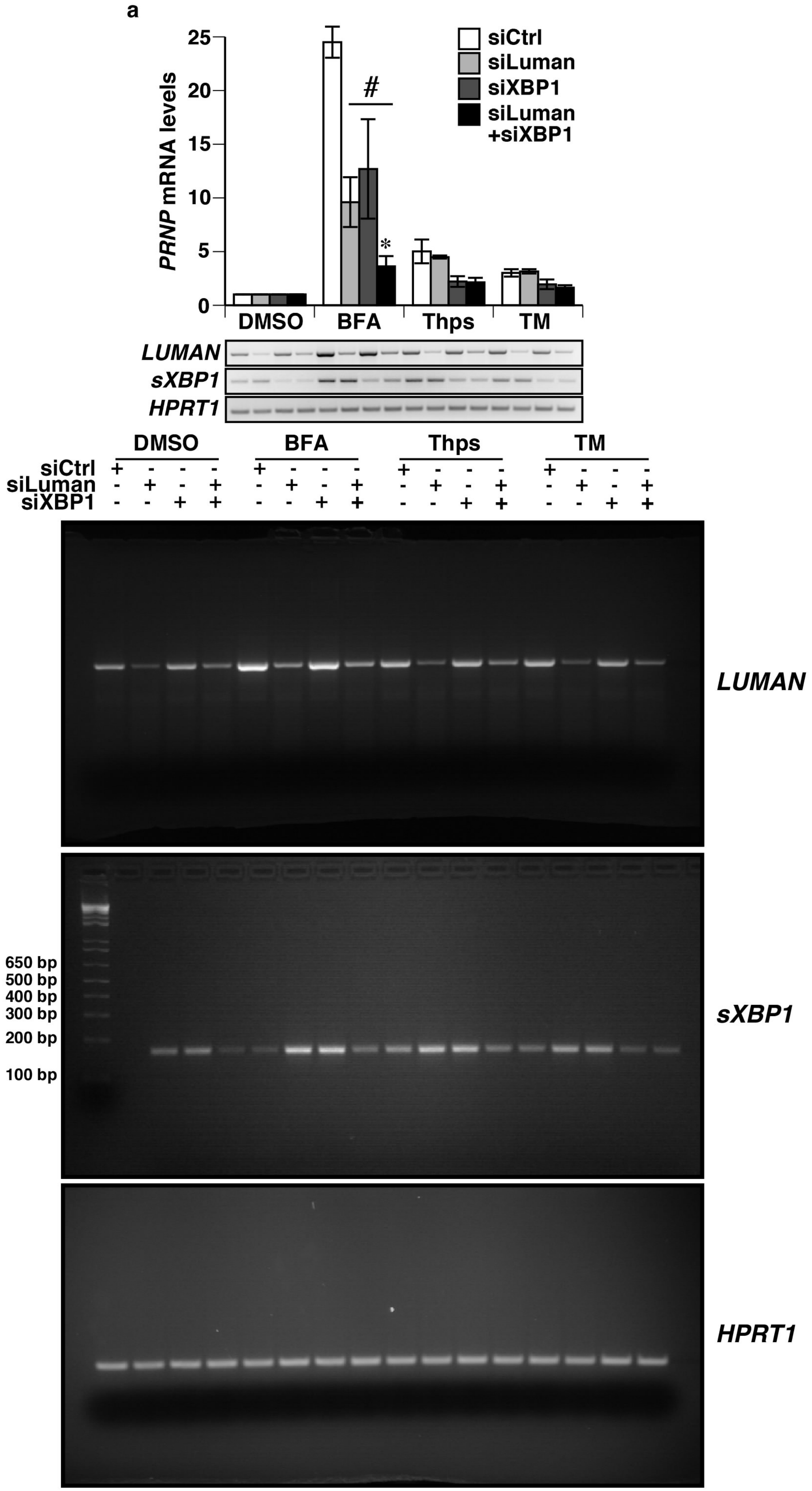
a
PRNP mRNA levels
0
5
10
15
20
25
siCtrl
siLuman
siXBP1
siLuman +siXBP1
#
*
DMSO
BFA
Thps
TM
LUMAN
sXBP1
HPRT1
siCtrl
siLuman
siXBP1
650 bp
500 bp
400 bp
300 bp
200 bp
100 bp
LUMAN
sXBP1
HPRT1

Figure 4b

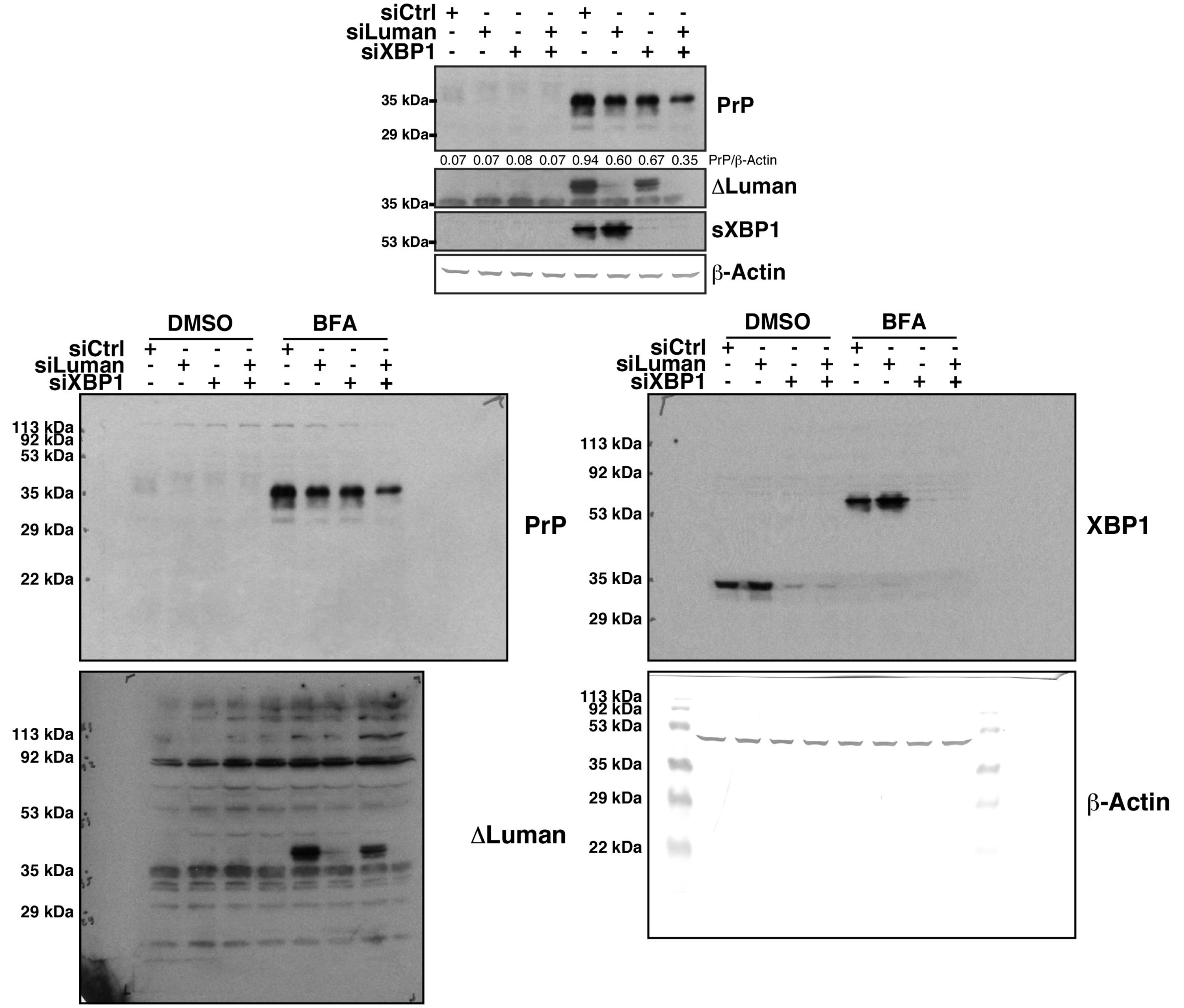

b
DMSO
BFA
siCtrl
siLuman
siXBP1
35 kDa
29 kDa
PrP
0.07 0.07 0.08 0.07 0.94 0.60 0.67 0.35
PrP/β-Actin
ΔLuman
35 kDa
sXBP1
53 kDa
β-Actin
113 kDa
92 kDa
53 kDa
35 kDa
29 kDa
22 kDa
PrP
XBP1
ΔLuman
β-Actin

Figure 4c

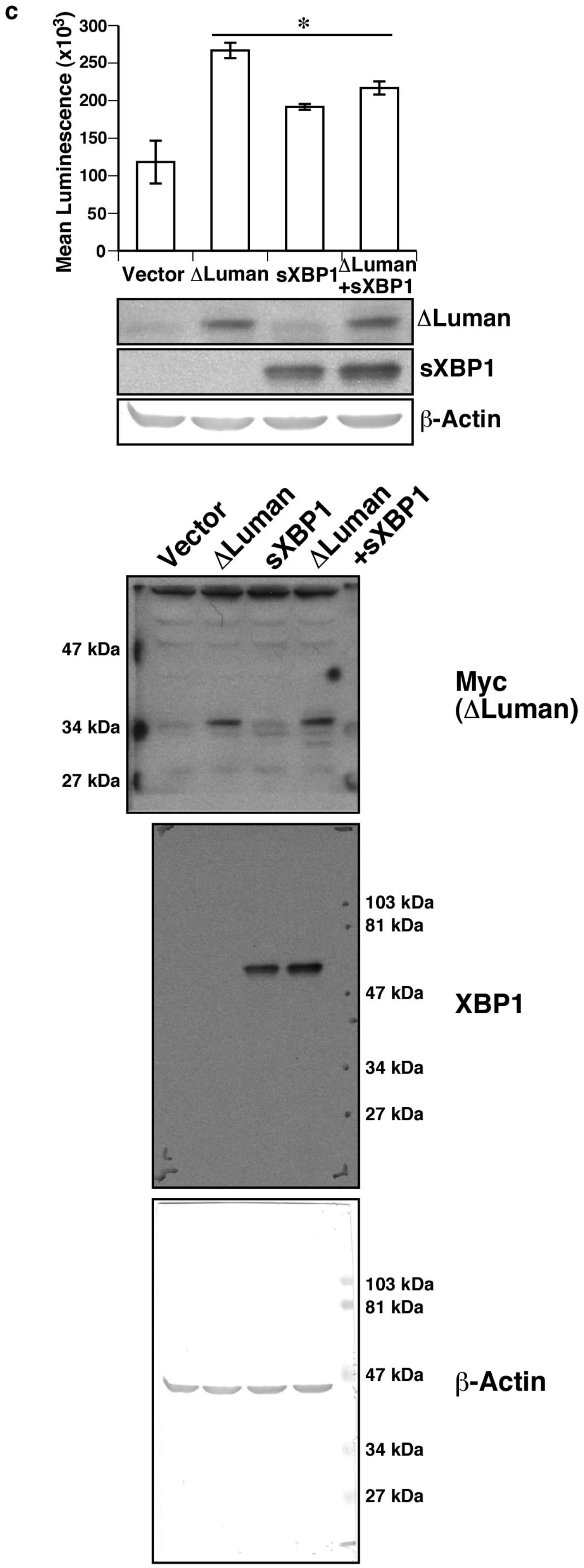
c
Mean Luminescence (x10^3)
300
250
200
150
100
50
0
*
Vector
ΔLuman
sXBP1
ΔLuman +sXBP1
ΔLuman
sXBP1
β-Actin
Vector
ΔLuman
sXBP1
ΔLuman +sXBP1
47 kDa
34 kDa
27 kDa
Myc (ΔLuman)
103 kDa
81 kDa
47 kDa
34 kDa
27 kDa
XBP1
103 kDa
81 kDa
47 kDa
34 kDa
27 kDa
β-Actin

Figure 5a

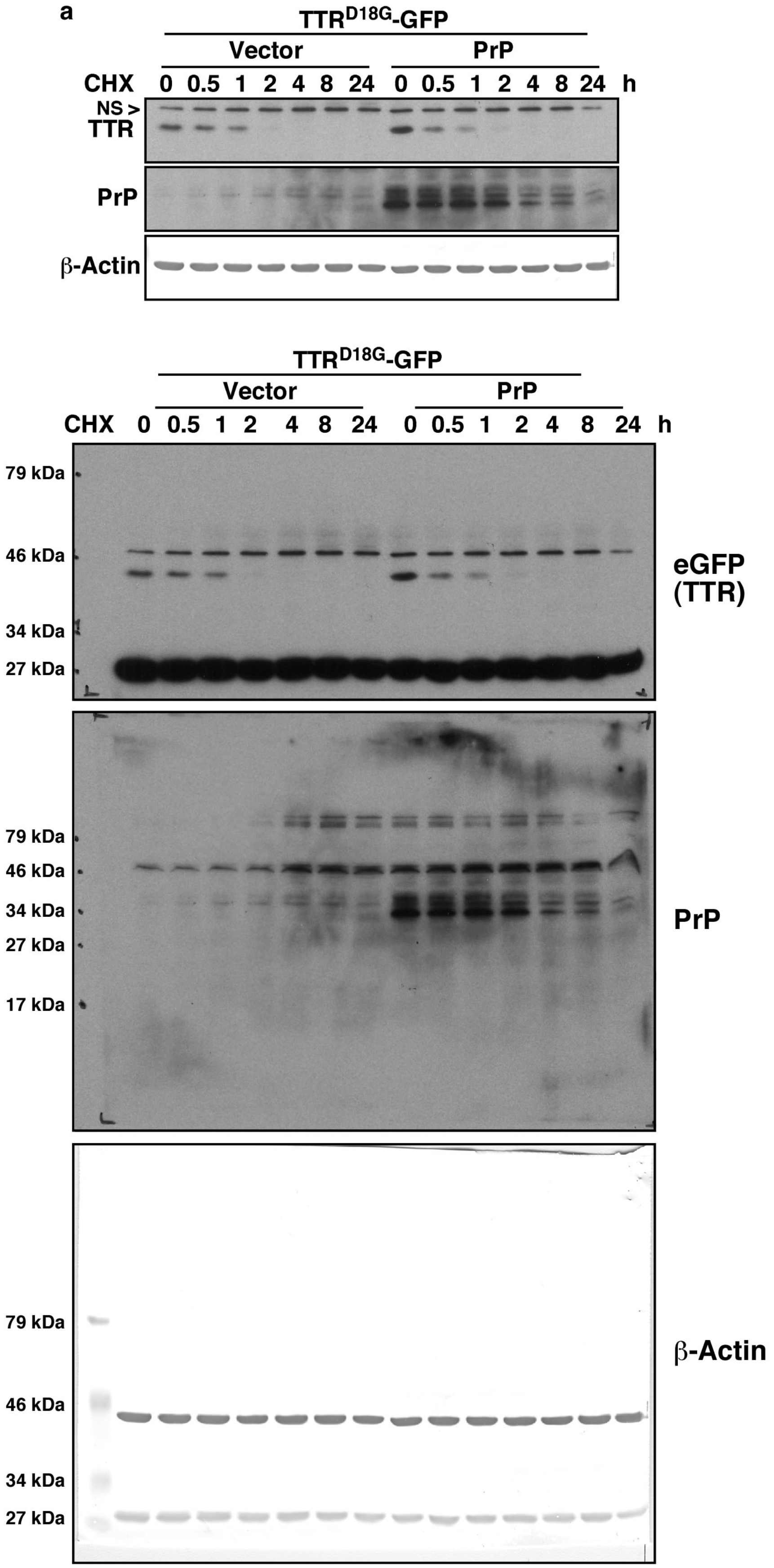
a
TTRD18G-GFP
Vector
PrP
CHX 0 0.5 1 2 4 8 24 0 0.5 1 2 4 8 24 h
NS >
TTR
PrP
β-Actin
TTRD18G-GFP
Vector
PrP
CHX 0 0.5 1 2 4 8 24 0 0.5 1 2 4 8 24 h
79 kDa
46 kDa
34 kDa
27 kDa
eGFP (TTR)
79 kDa
46 kDa
34 kDa
27 kDa
17 kDa
PrP
79 kDa
46 kDa
34 kDa
27 kDa
β-Actin

Figure 5c

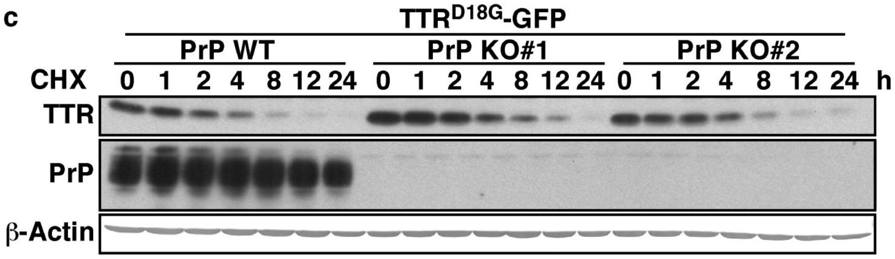
c
TTR^{D18G}-GFP
PrP WT
PrP KO#1
PrP KO#2
CHX 0 1 2 4 8 12 24 0 1 2 4 8 12 24 0 1 2 4 8 12 24 h
TTR
PrP
β-Actin

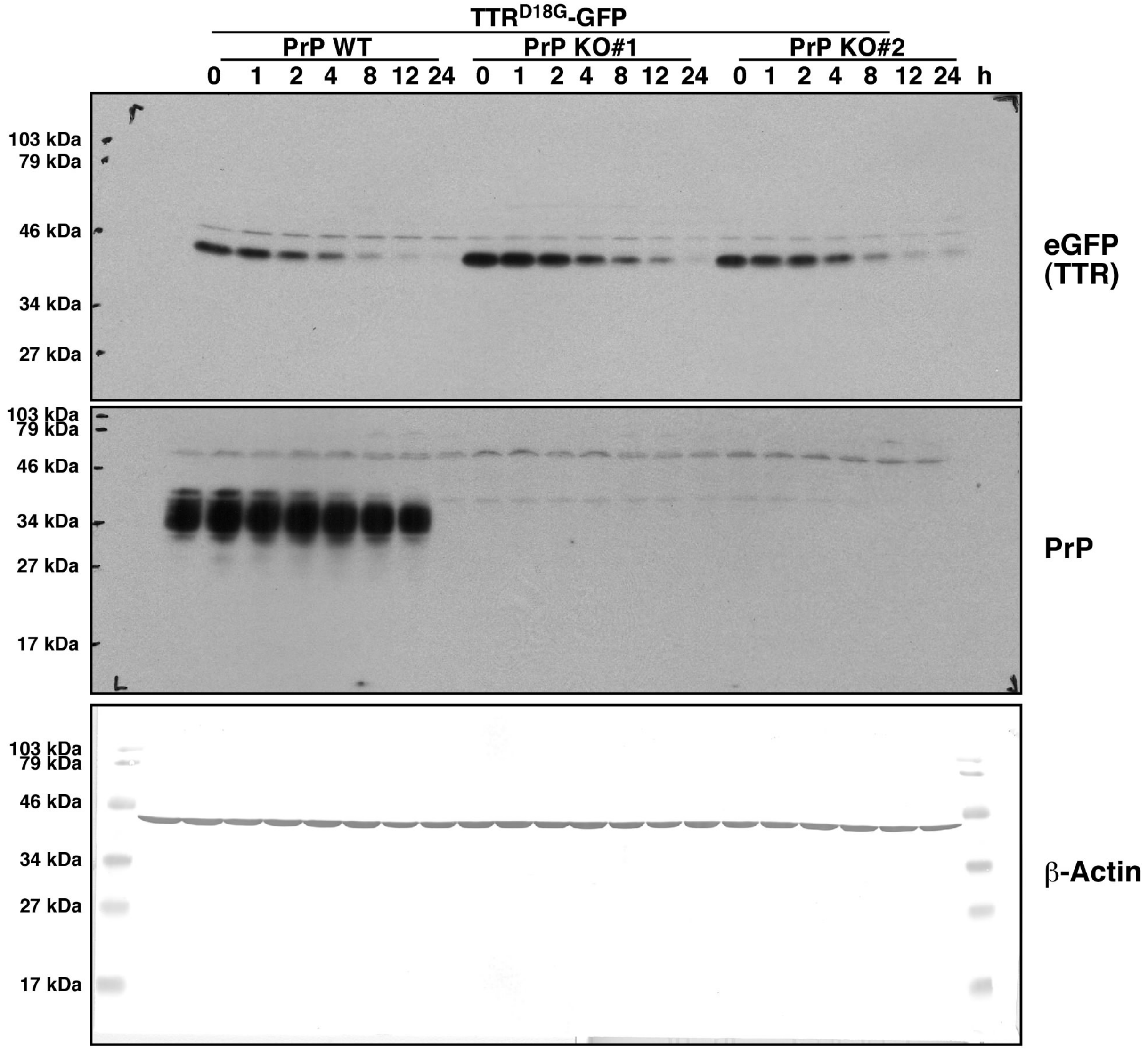
TTR^{D18G}-GFP
PrP WT
PrP KO#1
PrP KO#2
0 1 2 4 8 12 24 0 1 2 4 8 12 24 0 1 2 4 8 12 24 h
103 kDa
79 kDa
46 kDa
34 kDa
27 kDa
eGFP (TTR)
103 kDa
79 kDa
46 kDa
34 kDa
27 kDa
17 kDa
PrP
103 kDa
79 kDa
46 kDa
34 kDa
27 kDa
17 kDa
β-Actin

Figure 6b

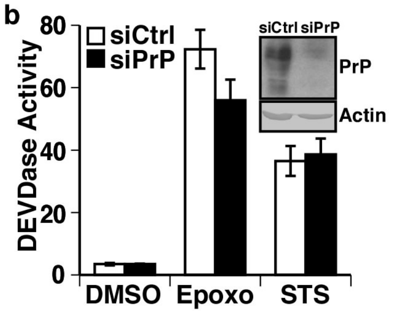
b
siCtrl
siPrP
DEVDase Activity
80
60
40
20
0
DMSO
Epoxo
STS
siCtrl siPrP
PrP
Actin

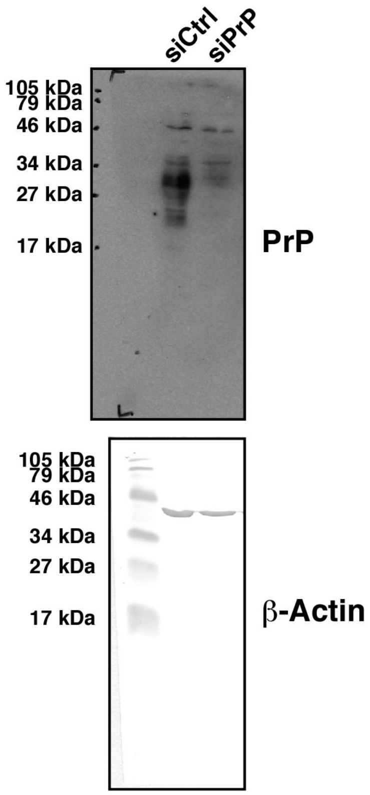
siCtrl
siPrP
105 kDa
79 kDa
46 kDa
34 kDa
27 kDa
17 kDa
PrP
105 kDa
79 kDa
46 kDa
34 kDa
27 kDa
17 kDa
β-Actin

Figure 7b

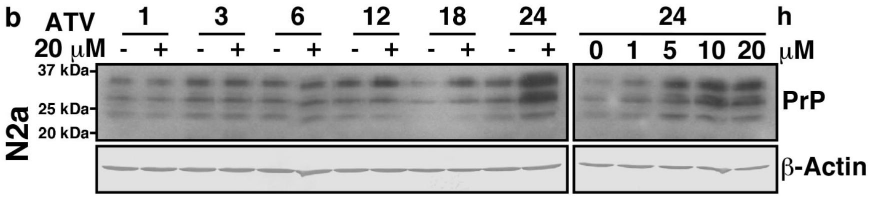

b
ATV
20 μM
1 3 6 12 18 24
- + - + - + - + - + - +
24 h
0 1 5 10 20 μM
37 kDa
25 kDa
20 kDa
N2a
PrP
β-Actin

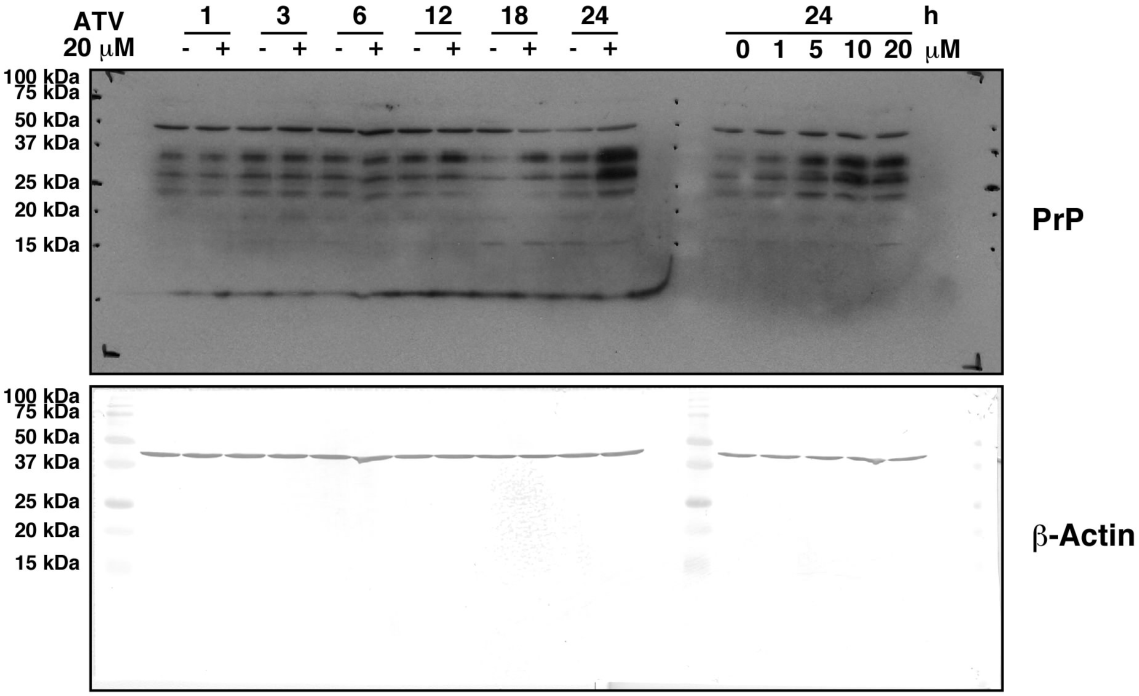

ATV
20 μM
1 3 6 12 18 24
- + - + - + - + - + - +
24 h
0 1 5 10 20 μM
100 kDa
75 kDa
50 kDa
37 kDa
25 kDa
20 kDa
15 kDa
PrP
100 kDa
75 kDa
50 kDa
37 kDa
25 kDa
20 kDa
15 kDa
β-Actin

Figure 7c

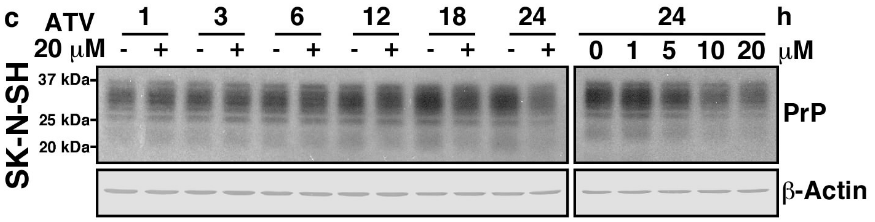
c
ATV
1
3
6
12
18
24
24
h
20 μM
- + - + - + - + - + - +
0 1 5 10 20 μM
SK-N-SH
37 kDa
25 kDa
20 kDa
PrP
β-Actin

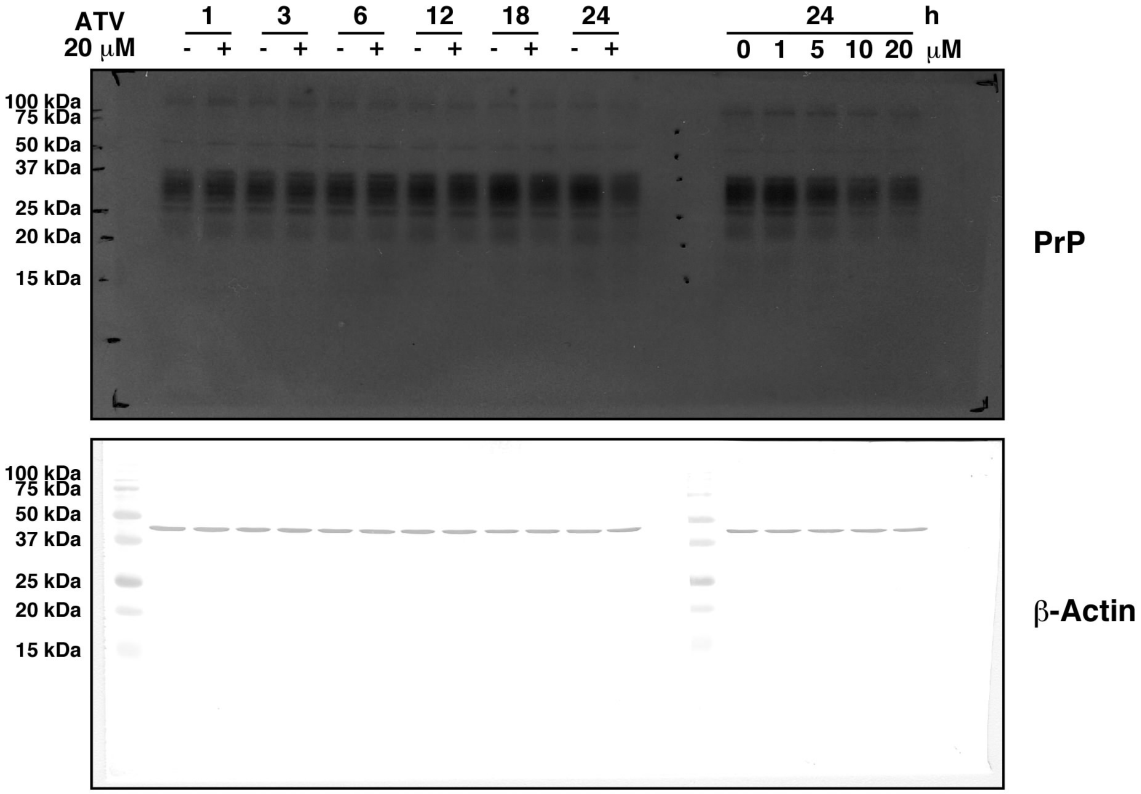
ATV
1
3
6
12
18
24
24
h
20 μM
- + - + - + - + - + - +
0 1 5 10 20 μM
100 kDa
75 kDa
50 kDa
37 kDa
25 kDa
20 kDa
15 kDa
PrP
100 kDa
75 kDa
50 kDa
37 kDa
25 kDa
20 kDa
15 kDa
β-Actin

Figure 7e

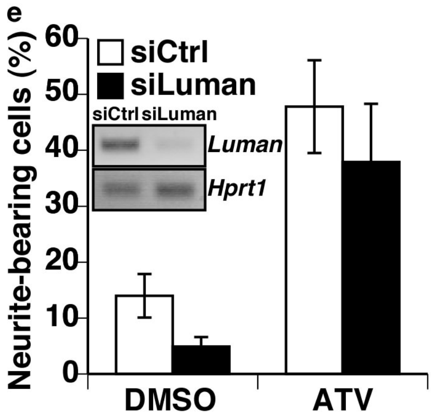
e
Neurite-bearing cells (%)
60
50
40
30
20
10
0
siCtrl
siLuman
siCtrl siLuman
Luman
Hprt1
DMSO
ATV

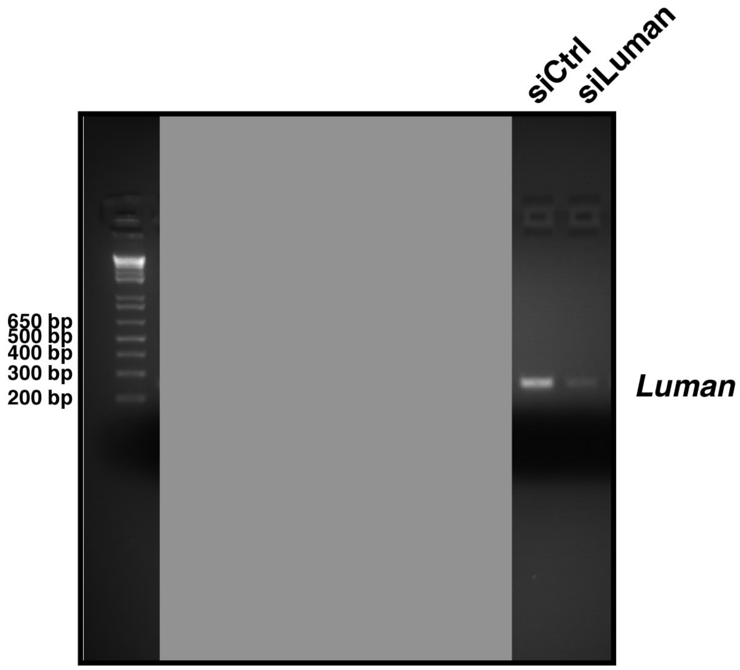
siCtrl
siLuman
650 bp
500 bp
400 bp
300 bp
200 bp
Luman

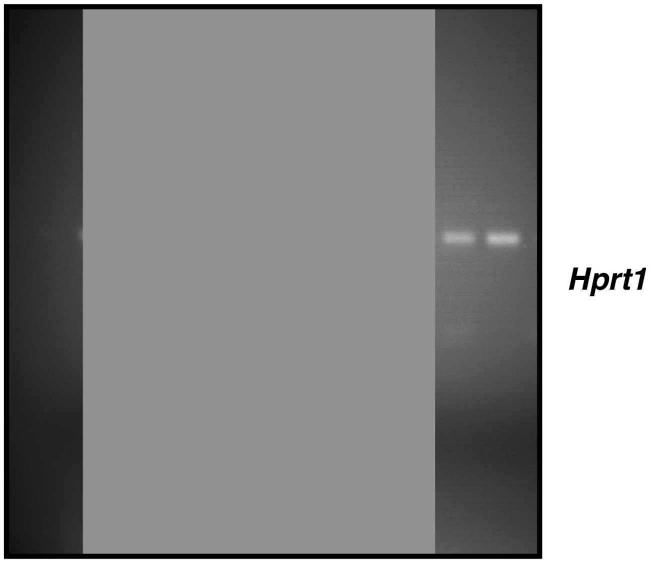
Hprt1

Figure 7g

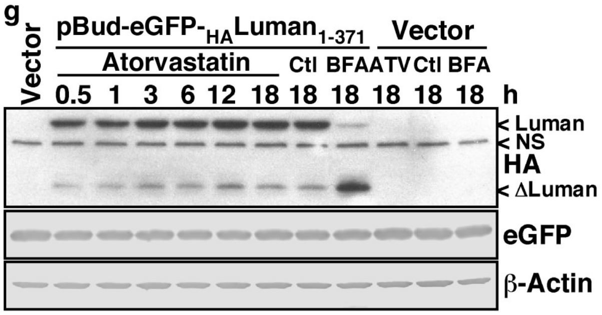
g
Vector
pBud-eGFP-HALuman1-371
Vector
Atorvastatin
Ctl
BFA
ATV
Ctl
BFA
0.5 1 3 6 12 18 18 18 18 18 18 h
Luman
NS
HA
ΔLuman
eGFP
β-Actin

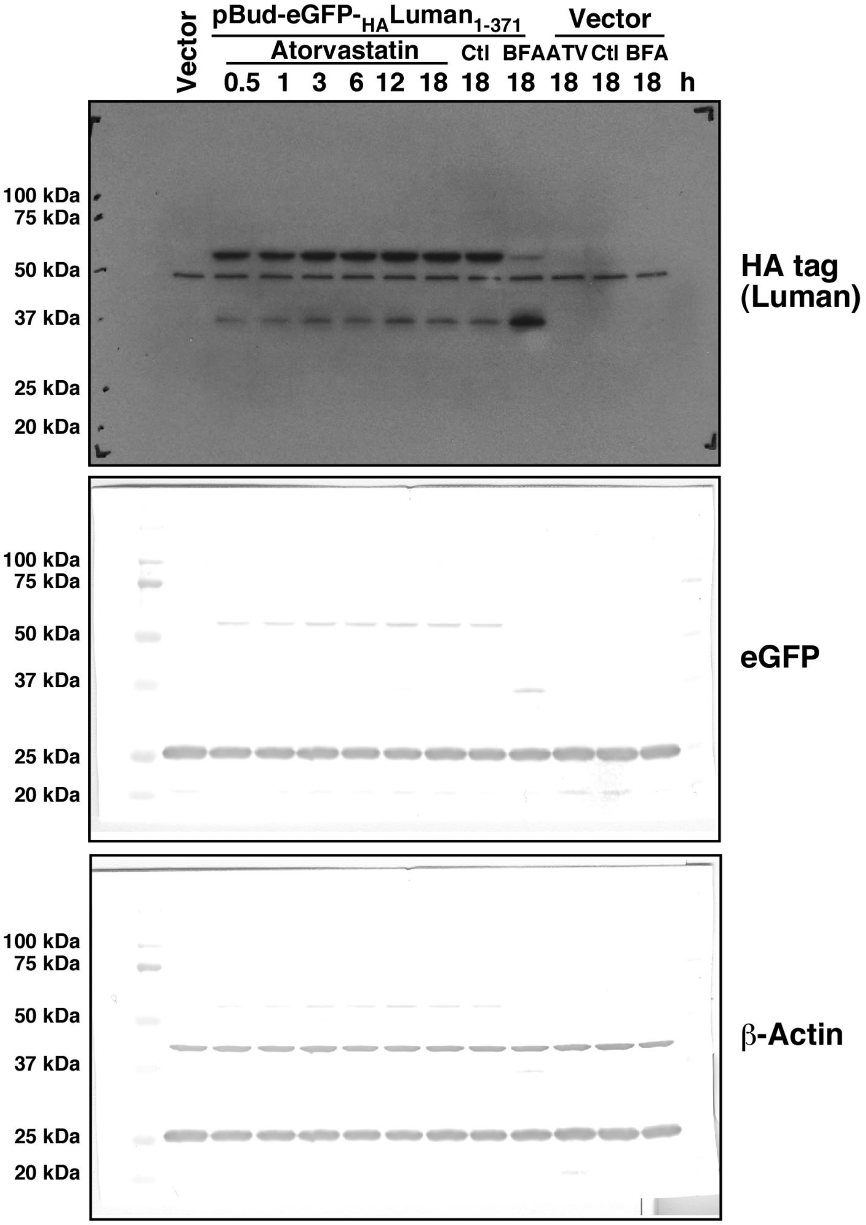
Vector
pBud-eGFP-HALuman1-371
Vector
Atorvastatin
Ctl
BFA
ATV
Ctl
BFA
0.5 1 3 6 12 18 18 18 18 18 18 h
100 kDa
75 kDa
50 kDa
37 kDa
25 kDa
20 kDa
HA tag
(Luman)
eGFP
β-Actin

Figure 7h

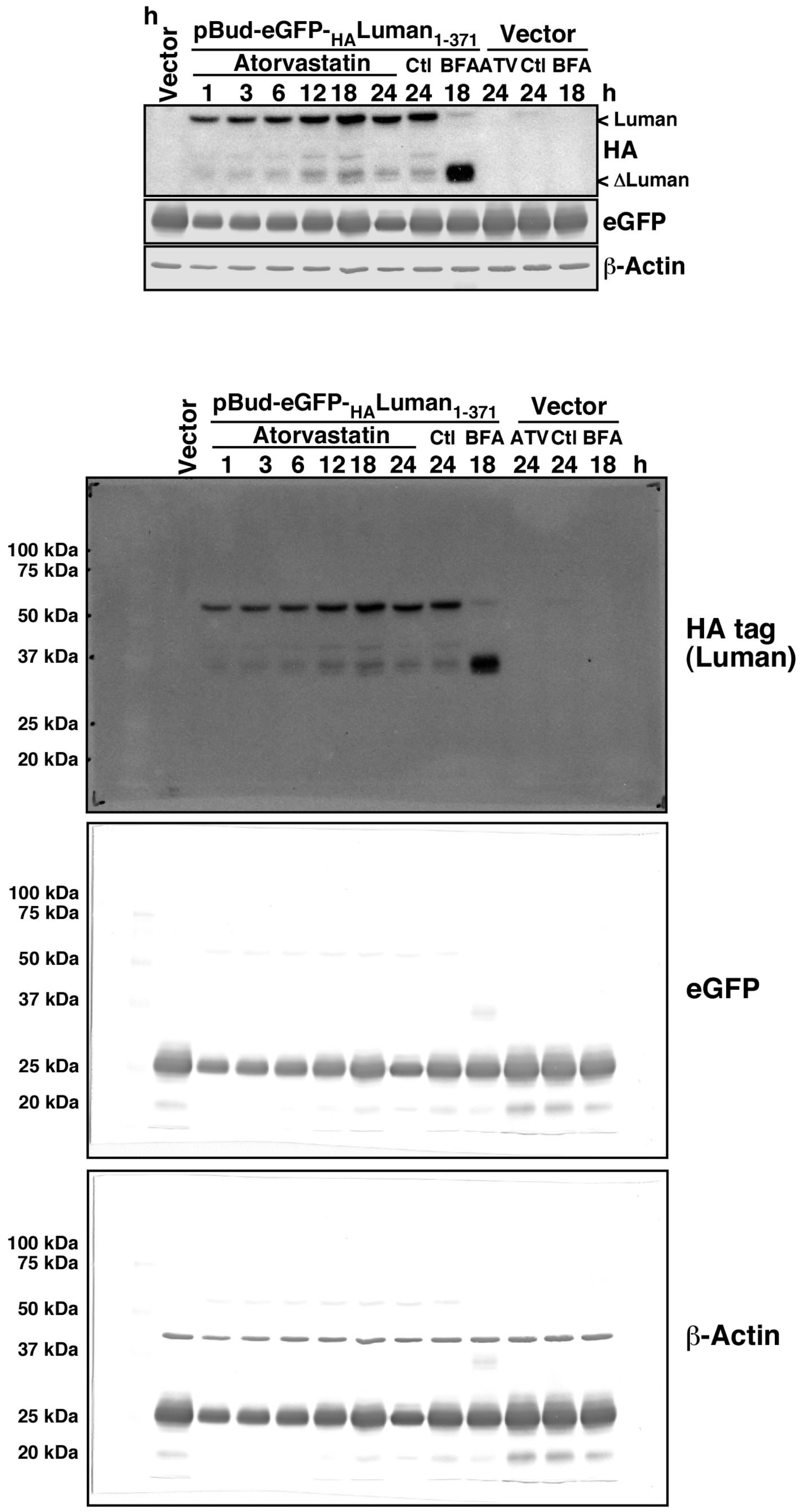
h
Vector
pBud-eGFP-HALuman1-371
Vector
Atorvastatin
Ctl
BFA
ATV
Ctl
BFA
1 3 6 12 18 24 24 18 24 24 18 h
< Luman
HA
< ΔLuman
eGFP
β-Actin
Vector
pBud-eGFP-HALuman1-371
Vector
Atorvastatin
Ctl
BFA
ATV
Ctl
BFA
1 3 6 12 18 24 24 18 24 24 18 h
100 kDa
75 kDa
50 kDa
37 kDa
25 kDa
20 kDa
HA tag
(Luman)
100 kDa
75 kDa
50 kDa
37 kDa
25 kDa
20 kDa
eGFP
100 kDa
75 kDa
50 kDa
37 kDa
25 kDa
20 kDa
β-Actin
